# Technical fishway passage structures provide high passage efficiency and effective passage for adult Pacific salmonids at eight large dams

**DOI:** 10.1371/journal.pone.0256805

**Published:** 2021-09-02

**Authors:** Matthew L. Keefer, Michael A. Jepson, Tami S. Clabough, Christopher C. Caudill

**Affiliations:** Department of Fish and Wildlife Sciences, College of Natural Resources, University of Idaho, Moscow, Idaho, United States of America; University of Waikato, NEW ZEALAND

## Abstract

Fishways have been widely used for upstream passage around human-built structures, but ‘success’ has varied dramatically. Evaluation of fishway success has typically been conducted at local scales using metrics such as fish passage efficiency and passage time, but evaluations are increasingly used in broader assessments of whether passage facilities meet population-specific conservation and management objectives. Over 15 years, we monitored passage effectiveness at eight dams on the Columbia and Snake rivers for 26,886 radio-tagged spring-summer and fall Chinook Salmon *O*. *tshwaytscha*, Sockeye Salmon *O*. *nerka*, and summer steelhead *O*. *mykiss* during their migrations to spawning sites. Almost all fish that entered dam tailraces eventually approached and entered fishways. Tailrace-to-forebay passage efficiency estimates at individual dams were consistently high, averaging 0.966 (*SD* = 0.035) across 245 run×year×dam combinations. These estimates are among the highest recorded for any migratory species, which we attribute to the scale of evaluation, salmonid life history traits (e.g., philopatry), and a sustained adaptive management approach to fishway design, maintenance, and improvement. Full-dam fish passage times were considerably more variable, with run×year×dam medians ranging from 5–65 h. Evaluation at larger scales provided evidence that fishways were biologically effective, e.g., we observed rapid migration rates (*medians* = 28–40 km/d) through river reaches with multiple dams and estimated fisheries-adjusted upstream migration survival of 67–69%. However, there were substantive uncertainties regarding effectiveness. Uncertainty about natal origins confounded estimation of population-specific survival and interpretation of apparent dam passage ‘failure’, while lack of post-migration reproductive data precluded analyses of delayed or cumulative effects of passing the impounded system on fish fitness. Although the technical fishways are effective for salmonids in the Columbia-Snake River system, other co-migrating species have lower passage rates, highlighting the need for species-specific design and evaluation wherever passage facilities impact fish management and conservation goals.

## Introduction

Fishways and other engineered fish passage structures are widely used to mitigate the negative effects of dams in rivers with diadromous and resident fish populations. Unfortunately, many fish passage projects have been ineffective for both economically valuable target species [1–3] and for broader native species assemblages worldwide [4–6]. Declines and extirpations of river fish populations have accelerated in parallel with river development [7–11], and the ecological and socioeconomic importance of improving river connectivity for fish and other riverine species has never been greater, with increasing risk [12–14] and species extinctions [15]. The many challenges for river-dependent species have spurred a surge in technical reviews of fish passage design [16–19], fishway and dam passage efficiency [1, 20–22], and the broader biological effectiveness of fish passage projects [20, 23–25].

Some of the more successful fish passage facilities have been preceded by a design and testing process featuring extensive collaboration among engineers and biologists. A particularly intensive example was the development of fishways for adult Pacific salmonids (*Oncorhynchus* spp) that coincided with the 20^th^ Century construction of large hydroelectric dams in the Columbia River Basin [CRB; 26]. The high cultural and economic value of anadromous salmonids in the Pacific Northwest provided sufficient incentive for an integrated research and development program, which has included hydraulic modeling of various fishway designs, construction of full-scale fishway models, and–importantly–tests using native salmonid fishes from the affected populations [26]. The swimming capabilities of adult salmonids and their philopatric life history were integral to the success of the CRB fishway design program. The high aerobic capacity and burst swim speeds of adult salmonids [27, 28] were well matched to the pool-and-weir fishways [18] that were eventually built throughout the CRB. Natal homing greatly increased the likelihood that adult migrants from upstream populations were motivated to search for and use the fishways at dams. The iterative fishway design and evaluation process, combined with the biological traits of the targeted species, has been credited with the success of upstream fish passage in the CRB. Hundreds of thousands of returning adult salmonids have passed dams each year over the last several decades [29].

The relatively high success of adult salmonids at fishways is often referenced as a gold standard for upstream fish passage. As a result, the pool-and-weir design deployed in the CRB has been exported around the world, often with little or no evaluation of its suitability for other endemic species. There are abundant examples of ‘salmonid style’ fishways that are ineffective for other fish communities [e.g., 5, 30, 31]. For that matter, fishways are not universally effective for salmonids. In their review, Noonan et al. [1] reported mean upstream passage efficiency was ~62% for 31 salmonid studies at a mix of fishway types and ~71% at pool-and-weir fishways specifically. Lower than desired salmonid passage success at fishways has frequently been attributed to confounding hydraulic conditions, such as low fish attraction flow from fishway entrances or misdirected attraction to spillway or turbine discharge, or to behavioral barriers presented by high velocity or turbulence [2, 32–35].

The recent fishway and dam passage reviews have included surprisingly few estimates from the data-rich CRB, and addressing that deficiency is a principal objective of this paper. Over the last two decades, there has been a multi-agency CRB research program for adult Chinook Salmon (*O*. *tshawytscha*), Sockeye Salmon (*O*. *nerka*), and steelhead (anadromous *O*. *mykiss*). The adaptive management program was motivated by steep 20^th^ Century declines in salmon and steelhead abundance and subsequent U.S. Endangered Species Act protections for many CRB populations [36, 37]. Adult salmonid migration research topics have ranged from narrowly-defined questions about how CRB fishway structures or dam operations affect fish behaviors at project-specific spatial scales [e.g., 38–42], to system-wide evaluations of upstream migration rates [43, 44] survival [45, 46] and homing success [47, 48]. Radiotelemetry has been the primary technological tool in the CRB adult salmonid program, and the large datasets have also been used to investigate broader ecological questions. For example, we evaluated the impacts of an influx of sea lions (*Zalophus califonianus*, *Eumetopias jubatus*) in the lower Columbia River [49, 50] and how warm river conditions affect the upstream migration behavior and success of migrants [e.g., 51–54]. The data have not been used to systematically report fishway and dam passage efficiency or effectiveness in the peer-reviewed literature.

Herein we address three specific objectives using data collected from radio-tagged adult salmon and steelhead at eight Columbia and Snake River dams. First, we summarize fish passage efficiency metrics (i.e., proportion-based metrics of upstream passage ‘success’, [1, 20, 25]) through four segments at the dams. The metrics are: (1) fishway attraction efficiency, from tailrace entry to approach a fishway opening; (2) fishway entrance efficiency, from fishway approach to fishway entry; (3) fishway passage efficiency, from fishway entry to pass a dam; and (4) dam passage efficiency, i.e., full-project passage efficiency from tailrace arrival past a dam. Second, we summarize fish passage times, an important component of assessing overall dam passage effectiveness [44, 55, 56], through the tailrace, fishway, and full-project segments, as well as past multiple dams and reservoirs to put fish passage at single dams into a broader migration context. Third, we summarize the final distribution of all radio-tagged fish because assessing fish passage effectiveness also requires an understanding of fish distribution and fate after they leave a facility [20, 23, 55]. Of particular interest in the fate assessment were the groups of radio-tagged fish that approached but ultimately did not pass individual dams, a behavior that could be attributed to a variety of factors ranging from actual passage failure, to downstream harvest, to imprecise homing movements by fish from downstream spawning sites.

## Methods

### Ethics statement

The methods used to collect, tag, and monitor adult salmon and steelhead were approved by the University of Idaho Animal Care and Use Committee, and are summarized in the “Fish collection and Selection”, “Fish Assessment, Tagging, and Release”, and “Telemetry Monitoring” sections below. Fish collection was permitted annually by NOAA-Fisheries (e.g., Research Action 994) and the State of Washington (e.g., Scientific Collection Permit 02–150).

### Study area

**Fig 1:**
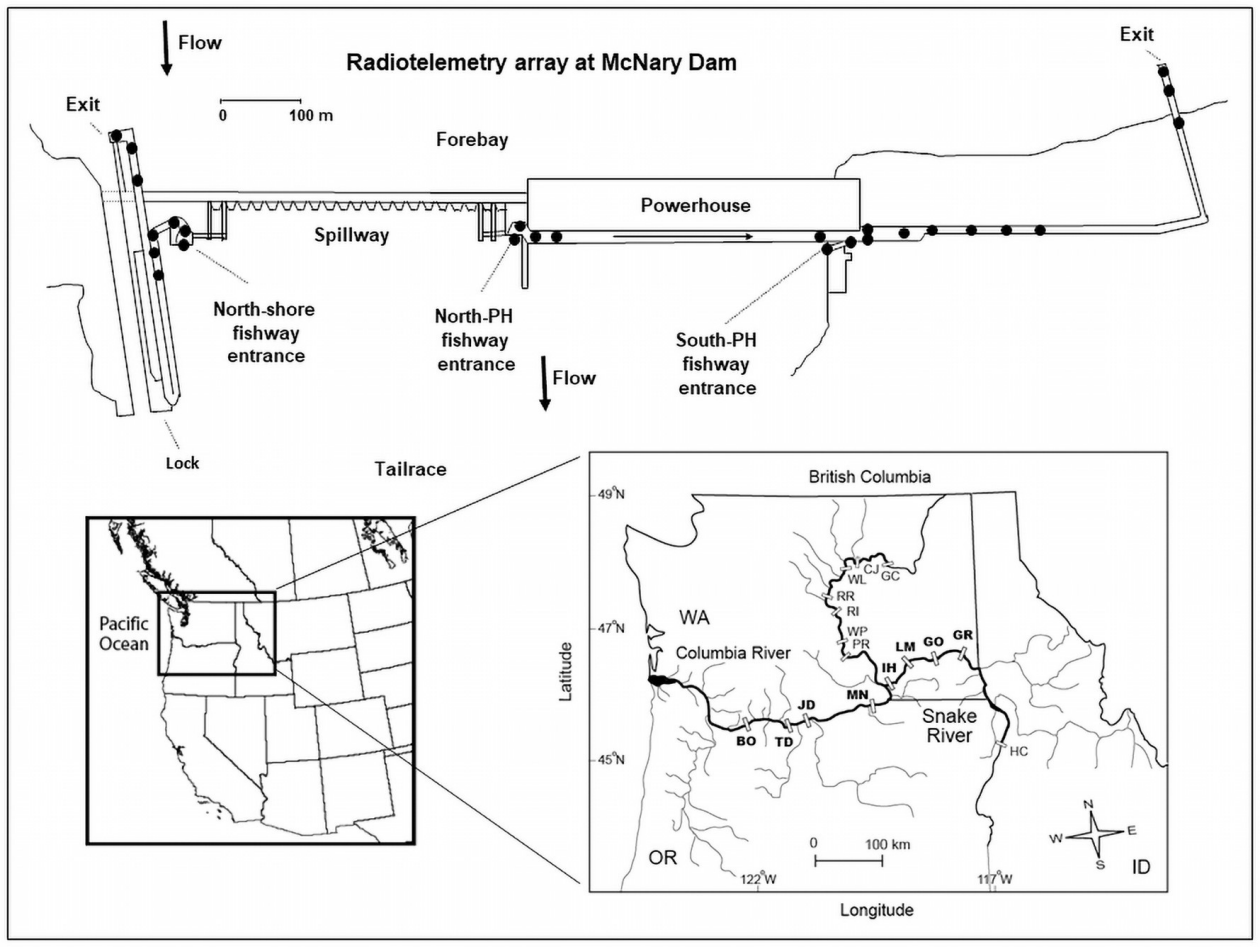
Diagram showing a typical radiotelemetry-monitoring array used in annual adult salmon and steelhead studies. The example shows McNary Dam (MN) on the lower Columbia River and includes the primary adult fishway entrances and sites (●) where single or multiple underwater coaxial cable antennas were used to monitor fish movements; aerial antennas located downstream to monitor the dam tailrace are not shown. Insets show the western United States and Canada (left) and the locations of dams in the lower Columbia and Snake Rivers (right). Bold text indicates study dams: BO (Bonneville), TD (The Dalles), JD (John Day), MN (McNary), IH (Ice Harbor), LM (Lower Monumental), GO (Little Goose), and GR (Lower Granite). Dams that were not a part of this summary, but are mentioned in the text include: PR (Priest Rapids), WP (Wanapum), RI (Rock Island), RR (Rocky Reach), WL (Wells), CJ (Chief Joseph), GC (Grand Coulee), and HC (Hells Canyon); there is currently no upstream fish passage at Chief Joseph or Hells Canyon dams. The McNary Dam image is similar but not identical to line drawings by the U.S. Army Corps of Engineers and is for illustrative purposes only.

The Columbia River drains >660,000 km^2^ of seven U.S. states and two Canadian provinces (Fig 1) and has mean annual discharge >7,000 m^3^/s [57]. The basin historically supported some of the largest and most diverse Pacific salmon and steelhead runs in the world [58–60]. The Columbia River and its largest tributary, the Snake River, were transformed by hydropower development in the 20^th^ Century [61]. There are currently 14 dams and reservoirs on the main stem Columbia River (11 in the U.S. and 3 in Canada) and 20 on the main stem Snake River. Thirteen of these projects have fish passage facilities for upstream migrants. The Columbia River dams with adult fishways are: Bonneville (~235 river kilometers [rkm] from the Pacific Ocean), The Dalles (~308), John Day (~347), McNary (~470), Priest Rapids (~639), Wanapum (~669), Rock Island (~730), Rocky Reach (~762), and Wells (~830). The Snake River dams with fishways are: Ice Harbor (rkm ~538), Lower Monumental (~589), Little Goose (~635), and Lower Granite (~695).

We monitored adult salmon and steelhead at eight dams, four each on the lower Columbia and lower Snake rivers (Table 1). All eight dams are run-of-river projects with limited reservoir storage. At each project, upstream migrants must pass through turbulent, high-velocity (up to ~8 m/s) tailraces [62, 63], locate fishway openings near powerhouses or adjacent to spillways, and then swim up through fish ladders that rise ~17–31 m (Table 1). The number, types, and discharge volumes of fishway openings differ among projects, but all have a target hydraulic head of ~0.30 m and opening configurations to achieve near-surface attraction velocities of ~1.5–3.0 m/s [64, 65]. Inside the fishways, the configuration of collection channel (target velocity ~0.45–1.2 m/s), transition areas, junction areas, fish ladders, and fish counting stations are each unique. However, all eight dams have pool-and-weir fishway ‘ladders’ [e.g., 18] that rise ~0.3 m per pool (slope 1:10 to 1:16) separated by overflow weirs with submerged-orifices. Several fishways also have flow-regulating elements in the upper-most sections composed of vertical-slot or tilting weirs [e.g., 26].

**Table 1:** Geographic locations and structural details of the four lower Columbia River and four lower Snake River study dams and their fishways. Year = year construction was completed. Also see Fig 1.

		Location	Length	Head^1^	Turbines	Capacity	Fishway Exits^2^	Openings^3^
Dam	Year	(rkm)	(m)	(m)	(*n*)	(MW)	(*n*)	(*n*)
Bonneville	1938	235	1,056	17	18	1,104	2	8
The Dalles	1957	308	2,693	24	22	2,080	2	4
John Day	1968	347	2,327	31	20	2,480	2	3
McNary	1953	470	2,245	22	14	1,120	2	3
Ice Harbor	1961	538	860	29	6	693	2	3
L. Monumental	1969	589	1,155	30	6	930	2	3
Little Goose	1970	635	809	30	6	930	1	3
Lower Granite	1975	695	980	30	6	930	1	3

^1^ Mean of monthly means (2017): http://www.nwd.wc.usace.army.mil/dd/nwdp/project_daily/webexec/rep

^2^ Top-of-ladder exits into dam forebay

^3^Primary fishway entry locations (see text)

### Study populations

We radio-tagged spring- and summer-run Chinook Salmon (15 years), fall-run Chinook Salmon (8 years), Sockeye Salmon (3 years), and summer-run steelhead (9 years). Sampled fish were broadly representative of the runs at large (see collection details below) and were therefore from dozens of genetically- and phenotypically-distinct Columbia River populations [66–68]. The tagged samples included fish from evolutionarily significant units (ESUs) and distinct population segments (DPSs) that are federally protected under the U.S. Endangered Species Act [36, 37, 69]. These included ‘threatened’ lower Columbia River spring Chinook Salmon [70], Snake River spring/summer and fall Chinook Salmon [71], Snake River, middle Columbia River, and upper Columbia River steelhead [72, 73], and ‘endangered’ upper Columbia River spring Chinook Salmon [74] and Snake River Sockeye Salmon [75]. The remaining sampled fish were from the several populations that do not have special protected status including: mid-Columbia River spring Chinook Salmon, Deschutes River summer-fall Chinook Salmon, upper Columbia River summer-fall Chinook Salmon, Okanogan River Sockeye Salmon, and Lake Wenatchee Sockeye Salmon. Individuals in all populations enter freshwater weeks (fall Chinook salmon), months (spring-summer Chinook Salmon; Sockeye Salmon) or up to a year (steelhead) prior to spawning.

### Fish collection and selection

All adult salmon and steelhead were collected at the Bonneville Dam adult fish facility adjacent to the Washington-shore fishway. A picket lead in the main ladder diverted fish up a false ladder into a large collection pool inside the facility. From the pool, fish ascended two false weirs into flumes that directed fish back to the main fish ladder upstream from the picket lead unless personnel activated a pneumatic gate that diverted fish into an anesthetic tank. The person selecting fish had ~1 sec to identify species and operate the gate. In some years, the operator was also alerted electronically if a fish had a previously-implanted passive integrated transponder (PIT) tag (see below).

Fish selection was random insomuch as possible given that the facility was located on one of several fishways and operated only during the day. Additional exceptions to random selection for some samples included: (1) selection for ‘upriver bright’ fall Chinook Salmon, a life-history group that spawns at main stem and tributary locations throughout the Columbia basin [76, 77]; (2) selection against sexually mature ‘Tule’ fall Chinook Salmon that primarily spawn in the Bonneville reservoir reach [78]; (3) selection against jack salmon (1-ocean males) and steelhead <50 cm; (4) selection against fish with large, visible injuries that penetrated the body cavity; and (5) year- and run-specific selection related to adult fish that had received PIT tags as juveniles. Existing PIT tags provided information on fish origin (i.e., natal streams and hatcheries) and juvenile outmigration timing and transportation history (e.g., whether they were collected for downstream barge transport; [79]. We selected for previously PIT-tagged fish in 2000–2004 to assess effects of juvenile transport on adult survival and straying [47, 48]. We selected against previously PIT-tagged fish in 2005–2014 to minimize potential effects on other concurrent studies that had tagged fish as juveniles. Very few previously PIT-tagged fish were collected in 1996–1998.

Adult fish were tagged throughout annual runs in approximate proportion to long-term average counts at Bonneville Dam. Variability in daily counts and annual run timing, as well as year-to-year differences in study objectives, precluded precisely proportional sampling. Factors that affected sample timing included: (1) block study designs related to dam operations; (2) animal care regulations restricting adult handling during warm-water periods; (3) annual study objectives that targeted spring Chinook Salmon [e.g., 50] or late-run steelhead [e.g., 46]; and (4) institutional issues related to facility operation and transmitter procurement.

### Fish assessment, tagging, and release

Fish selected for radio-tagging were diverted from the flumes into an anesthetic tank that contained Columbia River water and either 100 mg/L of tricaine methanesulfonate (MS-222), ~25 mg/L of clove oil (active ingredient: Eugenol), or ~18ml/L of AQUI-S 20 E (Aquatactics, Kirkland, Washington). Concentrations were adjusted based on water temperature and fish response. While fish were sedated, we recorded fish length, estimated sex based on body and head morphology, noted presence of hatchery fin clips, scanned fish for existing PIT tags, and recorded a variety of fish condition and injury metrics [e.g., 49]. In some years, fish were photographed, weighed, or had lipid levels assessed using a noninvasive Distell Fatmeter (Distell Industries, West Lothian, Scotland). Scales and a fin tissue sample were collected and archived in all years, but genetic samples were processed only in 2013–2014 [e.g., 46].

We inserted a glycerin-coated radio transmitter intra-gastrically into the stomach of each fish. A ~5-mm piece of latex surgical tubing was attached to most transmitters to reduce regurgitation rates [80]. The transmitter antenna was bent at the fish’s mouth and trailed alongside the body. We used ten radio transmitter models from the CART, LTD, MCFT, MST, and NTC series (Lotek Wireless, Inc., Newmarket, Ontario). Model changes reflect evolving technology, with a trend toward smaller batteries and smaller overall size for a given transmitter life. Transmitter lengths ranged from 2.6–9.0 cm, diameters ranged from 0.9–2.0 cm, and in-air weight ranged from 4.0–34.0 g. Larger transmitters were exclusively used in Chinook Salmon and large-bodied steelhead; smaller models were in Sockeye Salmon and small-bodied steelhead in all years and in all fish in some years. All transmitters were <2% of fish body weight as per [81]. A large majority of the transmitters had only a radio signal, but some had additional features such as depth and temperature sensors [e.g., 40, 54] or acoustic capabilities. From 1996–2009, there were 212 unique codes per channel on the 149 MHz frequency (149.320–149.800). Starting in 2010, the code set allowed 520 codes per channel on the 167 Mhz frequency (167.320–167.800). Signal transmission occurred every 4.5–5.5 seconds in all years. Secondary markers included a uniquely coded, alphanumeric visual implant tag inserted into the clear tissue posterior to the eye and a 1 mm-long coded wire tag inserted into the muscle near the dorsal fin (1996–2000), or a sterilized 12-mm PIT tag injected into the abdominal cavity (2000–2014). PIT tags were only implanted in adults that did not have an existing PIT tag.

After tagging, fish recovered in a 2,275-L tank filled with oxygenated river water. Upon regaining equilibrium, most fish (85.8%) were transported by truck to release sites on both sides of the river ~10 km downstream from Bonneville Dam. The remaining fish were transported to release sites in the dam forebay (10.8%) or were allowed to volitionally re-enter the Washington-shore fishway (3.4%) by passing over a weir (Table 2). Adult mortality during collection, tagging, and transport was <0.1%.

**Table 2:** Annual numbers and release locations of adult Chinook Salmon, Sockeye Salmon, and steelhead that were collected and radio-tagged at Bonneville Dam Adult Fish Facility (AFF) in 1996–2014.

		Spring-Summer	Fall	Sockeye	
Year	Release site	Chinook Salmon	Chinook Salmon	Salmon	Steelhead
1996	Downstream	853	**-**	**-**	769
1997	Downstream	1,014	55	577	975
1998	Downstream	957^1^	1,032	**-**	**-**
2000	Downstream	973	745	**-**	843
	Forebay	159	373	**-**	317
2001	Downstream	886^2^	561	**-**	804^3^
	Forebay	326	431	**-**	347
2002	Downstream	898	755	**-**	945
	Forebay	317	310	**-**	328
2003	Downstream	1,184^4^	666^5^	**-**	615
2004	Downstream	548	571	**-**	296
	Adult fish facility	8	35	**-**	4
2005	Downstream	96	**-**	**-**	**-**
	Adult fish facility	47	600	**-**	**-**
2006	Downstream	358	**-**	**-**	**-**
	Adult fish facility	22	**-**	**-**	**-**
2007	Downstream	307	**-**	**-**	**-**
	Adult fish facility	193	**-**	**-**	**-**
2009	Downstream	599	**-**	**-**	**-**
2010	Downstream	600	**-**	**-**	**-**
2013	Downstream	600	**-**	399	789
2014	Downstream	600	**-**	399	800
**All**	**All sites**	**11,545**	**6,134**	**1,375**	**7,832**

^1^ 1 released at AFF;

^2^ 4 at AFF;

^3^ 2 at AFF;

^4^ 1 at AFF;

^5^ 1 at AFF

### Telemetry monitoring

Radio monitoring arrays were deployed to address a diverse mix of study objectives that varied among years and locations. Monitoring effort was intensive, with typical arrays at the relatively small fishways at Snake River dams having >20 unique antenna sites [e.g, 39] to >80 antennas at the large and complex Bonneville Dam fishways [e.g., 82]. In most study years, multiple radio receivers (models SRX400 or SRX400A combined with a DSP500, Lotek Wireless) and antennas were deployed at each of the eight dams. In dam tailraces, aerial 9-element Yagi antennas located ~1–4 km downstream from the dams were used to monitor fish as they approached the projects. Underwater coaxial cable antennas were positioned near fishway entrances, exits, and inside various fishway segments to monitor fish as they approached, entered, and passed through fishways. Even with this effort, equipment costs and the scale of the fishways resulted in monitoring gaps; gaps were most common at low-volume fishway openings along powerhouse faces where installation access was challenging.

A number of additional radiotelemetry sites were used to help identify the fate and distribution of tagged fish. In all years, aerial antennas were deployed inside most large Columbia and Snake River tributaries with SRX400, SRX400A, or SRX600 receivers. In some years, aerial and/or underwater antennas were used at Columbia River dams upstream from the Snake River confluence, and aerial antennas were used in reservoir and unimpounded reaches of the main stem Columbia and Snake rivers.

The extensive PIT-tag monitoring infrastructure in the Columbia River basin provided a secondary source of movement and distribution data for the radio-tagged fish. PIT-tag detection data were collected by state, federal, and tribal agencies using antennas inside dam fishways and bypass facilities, in some tributaries, and at hatcheries and traps. The number and distribution of PIT-tag detection sites substantially increased over the course of our study. Detections at these locations were aggregated and archived by the Pacific States Marine Fisheries Commission and accessed by us via the Columbia Basin PIT Tag Information System (http://www.ptagis.org).

### Telemetry data processing

The radiotelemetry data were regularly downloaded from receivers and assembled into annual databases by experienced biologists and technicians. Pre-processing data quality control filters included removing likely ‘noise’ records created by signal collisions (i.e., two or more codes at the same receiver at the same time), removing records from channels or codes that had not been released in fish, and removing some records that did not have corroborating support from detections at nearby antennas.

Once the raw datasets were assembled and pre-screened, we used an evolving series of programs to code fish activities at the dams. In early study years, telemetry data were processed in ArcView (Esri, Redlands, California) using interactive maps of each fishway and vetted decision trees that prompted trained personnel to code selected records (typically the first and/or last records in a consecutive block of detections at a single antenna). In later years, a fully automated coding program was developed in Visual Basic (Microsoft, Redmond, Washington) that could be modified for each dam and each configuration of receivers and antennas. Examples of activity codes during both eras included when fish approached and entered fishways, exited a fishway into a tailrace, exited a ladder into a dam forebay, and passed individual antennas inside fishways. Codes also differentiated first events (e.g., first fishway entry) from subsequent events. An important component of the coding at dams in all years was the use of placeholder records when fish activities were inferred. Examples included ‘unknown’ fishway approaches and entries, which were assigned when a fish was detected inside a fishway but not at an antenna at a fishway opening, and ‘unknown’ dam passage events, when, for example, a fish was not detected on a top-of-ladder receiver while exiting but was later detected at an upriver location. Such coding allowed inclusion of many individuals with missing records when calculating efficiency metrics. Individuals with unknown start- or end-time events were censored from passage time calculations.

Coded records from all dams were integrated with several ancillary datasets, including: (1) detections at aerial antennas in reservoirs and tributaries; (2) records collected during truck- and boat-based mobile tracking; (3) PIT-tag detection records; (4) recapture records at hatchery traps and weirs; and (5) harvest records derived from a transmitter-return reward program (typically US$25–100 per tag, [46]). All data types were assembled chronologically and then further reduced by coding first and last detections from blocks of records at tributary and reservoir sites and from mobile surveys. The final quality control step was a review of each individual full-migration fish history by experienced personnel.

### Detection efficiency of monitoring arrays

Logistical and safety constraints prevented us from formally testing the detection range [e.g., 83] and *in situ* detection efficiency [e.g., 84] of most antennas. Boat access was restricted in large sections of the dam tailraces, including near fishway openings, and many fishway segments were inaccessible except when dewatered during winter maintenance periods. In several informal evaluations, we estimated detection ranges for the underwater coaxial cable antennas inside fishways to be ~5–15 m, sufficient to detect almost all fish passing through constricted segments (e.g., ladders) and most fish passing through deeper (e.g., some fishway openings) and wider (e.g., some transition areas) segments. The detection efficiency of individual fishway antennas was often > 90% and radio and PIT antenna redundancy inside fishways and upstream locations greatly reduced the likelihood of undetected fishway passage.

Rather than calculating antenna-specific detection efficiency, which was not particularly germane to estimating fish passage efficiency and passage time metrics, we calculated the proportion of radio-tagged fish in each run×year that were detected on their first passage event at four important transitions at each dam. These were: (1) the proportion detected at any radio antenna in a tailrace or at a dam that was detected on an aerial tailrace antenna on a fish’s presumed first passage through the tailrace; (2) the proportion detected at any antenna inside or outside a fishway that was detected on the presumed first approach at a fishway opening; (3) the proportion detected inside a fishway that was detected on the presumed first fishway entry; and (4) the proportion detected at a top-of-ladder antenna that was detected at any top-of-ladder site or upstream monitoring site.

First tailrace detection proportions were the lowest, on average, among the four metrics, with a mean of 0.735 (*SD* = 0.202, *n* = 247 run×year×dam combinations; S1 Fig in S2 Appendix). Missed first detections at the tailrace antennas were mostly attributable to the rapid attenuation of radio signals with fish depth [e.g., 84, 85] and to the bathymetry and width (often > 1 km) of tailrace channels. Detection proportions for first fishway entry events were also relatively low (*mean* = 0.747, *SD* = 0.178, *n* = 237; S2 Fig in S2 Appendix), with many missed events associated with fish use of unmonitored openings (e.g., many low-volume orifice and sluice gates along powerhouses). Detection proportions were higher and less variable for first fishway approaches (*mean* = 0.907, *SD* = 0.121, *n* = 235; S3 Fig in S2 Appendix) and top-of-ladder passage events (*mean* = 0.959, *SD* = 0.089, *n* = 223; S4 Fig in S2 Appendix). As with missed entries, missed first approaches were often at unmonitored openings, whereas missed detections at top-of-ladder sites typically occurred during power outages or other monitoring disruptions. We note that effective detection at the four locations where metrics were calculated were higher than the values above because many events could be inferred from detection at other locations at each dam or at upstream locations.

### Fish passage efficiency estimates

We quantified upstream passage success at dams using four attraction and passage metrics that are commonly defined as ‘efficiency’ metrics [1, 20, 30]. The proportion-based metrics were calculated using detection data from all similarly configured radiotelemetry sites at each dam to generate ‘dam-wide’ efficiency estimates [e.g., 82] because most dams had more than one fishway. As examples, detections at all fishway entrance antennas at a dam were combined and detections at all top-of-ladder exit antennas were combined when dams had more than one fishway. The metrics were:

1. Fishway attraction efficiency = (*N*_*Approach*_)×(*N*_*Tailrace*_ *+ N*_*Approach*_)^-1^
2. Fishway entrance efficiency = (*N*_*Enter*_)×(*N*_*Approach*_)^-1^
3. Fishway passage efficiency = (*N*_*Pass*_)×(*N*_*Enter*_)^-1^
4. Dam passage efficiency = (*N*_*Pass*_)×(*N*_*Tailrace*_ *+ N*_*Approach*_)^-1^

where *N*_*Tailrace*_ was the number of *unique* fish in a run×year that were detected on tailrace antennas; *N*_*Approach*_ was the number detected at radio antennas outside fishway openings or that were known to have approached based on detections inside fishways; *N*_*Enter*_ was the number of unique fish that were detected on radio antennas inside fishways; and *N*_*Pass*_ was the number of unique fish recorded at top-of-ladder radio antennas or that were inferred to have passed a dam from detections at upstream radiotelemetry sites or ladder PIT antennas. In the dam passage efficiency metric, *N*_*Pass*_ additionally included a small number of fish known to have passed a dam via a navigation lock, which were monitored at some dams in some years. Fish with no radio detections at a dam were excluded from all calculations, even if PIT data confirmed presence and/or passage. We also note that all fish were presumed naïve migrants (i.e., they had not experienced the fishways; [20]) at all dams upstream from Bonneville Dam; given the many passage routes at Bonneville Dam [82], many post-release fish were also naïve to the routes used there. Most fish approached, entered and used a single fishway at each dam, although some fish attempted to pass via multiple routes (i.e., switched fishways). We present dam-wide efficiency estimates to reduce the complicating effects of multiple passage routes and attempts by individuals, but also note that fishway-specific efficiency estimates tended to be similar at most individual dams.

### Fish passage times

As part of the effectiveness evaluation, we calculated fish passage times through essentially the same four transitions described for passage efficiency:

1. Tailrace passage time = (*App*_*F*_)—(*TR*_*F*_)
2. Fishway entrance time = (*Ent*_*F*_)—(*App*_*F*_)
3. Fishway passage time = (*Exit*_*L*_)—(*Ent*_*F*_)
4. Dam passage time = (*Exit*_*L*_)—(*TR*_*F*_)

where *TR*_*F*_ was the first detection on a tailrace antenna; *App*_*F*_ was the first detection on a radio antenna outside a fishway opening; *Ent*_*F*_ was the first detection on an antenna inside a fishway opening; and *Exit*_*L*_ was the last detection at a top-of-ladder fishway exit. Passage times through each segment potentially captured a wide variety of fish behaviors, including temporary downstream movement out of a tailrace (potentially including fallback at downstream dams), and movements between a tailrace and one or more fishways at a dam [e.g., 82, 86]. Passage time calculations were based solely on valid radio detections, (i.e., no placeholder records were used as start or end times). Consequently, some fish did not have times calculated for one or more passage segments.

### Final distribution

Final geographic location assignments for each fish were based on all available radiotelemetry, PIT-detection, reported harvest, and transmitter recovery data accumulated during the study [e.g., 45, 46]. Fish assigned to tributaries included those that were reported harvested in tributaries, entered hatcheries, were last detected on tributary radio or PIT antennas, or were recovered in tributary spawning ground surveys. Fish last detected in the main stem Columbia or Snake rivers included those that were reported harvested and those with unknown main stem fates. Most fish assigned unknown fate were last detected at antennas in tailraces or at dams and primarily represented presumed mortalities and apparent unreported harvest, rather than transmitter failure (based on concurrence of radio- and PIT telemetry records). The only excluded data were downstream movement records from presumed post-spawn fish (e.g., for steelhead kelts).

We used the final distribution data for two purposes. First, the proportions of fish from the full sample that entered spawning tributaries provided an indirect measure of dam passage effectiveness (i.e., the fish was able to complete migration after passing one or more dams, [20, 23]. Second, we assessed the final distribution of each subset of fish that approached–but ultimately did not pass–each of the eight dams. There are several potential reasons for non-passage, including some that are not directly related to the fishways, such as downstream predation, fisheries harvest, and the location of natal sites. Many adult salmon and steelhead detected at Columbia and Snake River dams, for example, have migrated upstream past their natal sites and must move back downstream to complete homing [e.g., 87, 88]. This natal site ‘overshoot’ behavior, as well as some harvest, should probably not be interpreted as dam passage failure.

## Results

### Radio-tagged samples

Over 15 years, we radio-tagged 11,545 spring–summer Chinook Salmon, 6,134 fall Chinook Salmon, 1,375 Sockeye Salmon, and 7,832 steelhead (Table 3). Fin clips were present on 47% (spring–summer Chinook Salmon), 9% (fall Chinook Salmon), 1% (Sockeye Salmon), and 68% (steelhead) of the tagged fish. Fin clipping was not standard protocol at all hatcheries in early study years, so hatchery-origin percentages were underestimated for some groups. Mean fork lengths for the four runs were 78.1 cm (*SD* = 8.3 cm, *range* = 47–117 cm), 81.5 cm (9.5, 50–125), 49.6 cm (3.8, 26–63) and 71.1 cm (9.9, 48–105), respectively. Sample sizes at the eight study dams declined from downstream to upstream (Table 3) as fish entered tributaries, were harvested, or were last detected in the migration corridor.

**Table 3:** Total and annual mean numbers of radio-tagged adult Chinook Salmon, Sockeye Salmon, and steelhead detected at the eight lower Columbia River and lower Snake River study dams, 1996–2014. The total released includes those released from the Bonneville Adult Fish Facility and into the Bonneville forebay. Sockeye Salmon detected at Snake River dams (≤ 3/year) not shown.

	Sp-Su Chinook		Fall Chinook		Sockeye		Steelhead	
	Total *n*	Mean *n*	Total *n*	Mean *n*	Total *n*	Mean *n*	Total *n*	Mean *n*
Total released^1^	11,545		6,134		1,375		7,832	
Below Bonneville	10,467		4,384		1,375		7,162	
Bonneville	10,245	683	4,135	591	1,338	446	6,667	741
The Dalles	9,222	615	4,521	565	1,222	407	6,344	705
John Day	7,991	533	3,597	450	1,164	388	5,665	629
McNary	7,154	477	2,779	347	1,129	376	4,980	553
Ice Harbor	3,606	240	376	47	-	-	3,830	426
L. Monumental	3,472	231	313	39	-	-	3,373	422
Little Goose	3,386	226	285	36	-	-	3,265	408
Lower Granite	3,295	220	259	32	-	-	3,414	379

^1^ Fish from all release sites combined; See Table 2 for details

### Fishway attraction efficiency

Almost all radio-tagged fish that entered dam tailraces eventually approached fishway openings (S5 Fig in S2 Appendix). Mean fishway attraction efficiency across all run×year×dam combinations was 0.985 (*SD* = 0.018, *n* = 223 combinations). Run-specific means varied only slightly at 0.988 (*SD* = 0.013) for spring–summer Chinook Salmon, 0.978 (0.028) for fall Chinook Salmon, 0.979 (0.013) for Sockeye Salmon, and 0.987 (0.011) for steelhead. The lowest mean estimates were at The Dalles Dam for spring–summer Chinook Salmon (0.976) and steelhead (0.980), at Ice Harbor Dam for fall Chinook Salmon (0.942), and at McNary Dam for Sockeye Salmon (0.977). Estimates were generally higher at Snake River dams than at Columbia River dams (S5 Fig in S2 Appendix).

### Tailrace passage time

Passage times through dam tailraces tended to be right-skewed, with longer times for fish that spent one or more nights in the tailrace or temporarily moved downstream before approaching a fishway (S5 Fig in S2 Appendix). Median times from first tailrace detection to first fishway approach were similar for all four runs, ranging from 2.2–2.7 h. The mean time for all run×year×dam combinations was 8.8 h (*SD* = 58.3 h, *n* = 69,481 individual times). Run-specific means were 8.3 h (*SD* = 35.1 h, *n* = 34,745) for spring–summer Chinook Salmon, 8.9 h (40.7, 9,150) for fall Chinook Salmon, 9.0 h (5.4, 3,569) for Sockeye Salmon, and 10.3 h (89.8, 22,017) for steelhead. The highest mean tailrace passage times were at Bonneville (spring–summer and fall Chinook Salmon), The Dalles (Sockeye Salmon), and Lower Granite (steelhead) dams. About 4.0% of all passage times through tailraces were ≥ 24 h and ~1.5% were ≥ 72 h (S5 Fig in S2 Appendix).

### Fishway entrance efficiency

Mean fishway entrance efficiency across all run×year×dam combinations was 0.990 (*SD* = 0.013, *n* = 238, S6 Fig in S2 Appendix). Run-specific means ranged from 0.985 (fall Chinook Salmon) to 0.995 (Sockeye Salmon and steelhead). The lowest mean estimates were at Bonneville Dam for spring–summer Chinook Salmon (0.983), Sockeye Salmon (0.987), and steelhead (0.991) and at Ice Harbor Dam for fall Chinook Salmon (0.964).

### Fishway entrance time

The time from first fishway approach to first entry was rapid for most fish at all dams (S6 Fig in S2 Appendix). Median times across dams were 1.9 h for spring–summer Chinook Salmon and ranged from 0.4–0.6 h for the other three runs. The mean time for all run×year×dam combinations was 8.5 h (*SD* = 51.3 h, *n* = 77,420). Run-specific means were 12.3 h (*SD* = 43.2 h, *n* = 35,743) for spring–summer Chinook Salmon, 4.0 h (26.6, 12,957) for fall Chinook Salmon, 3.6 h (5.4, 3,233) for Sockeye Salmon, and 6.2 h (70.6, 25,487) for steelhead. The highest mean fishway entry times were at Bonneville Dam for all runs except steelhead (Lower Granite Dam). About 6.0% of all first fishway entrance times were > 24 h and ~2.0% were > 72 h (S6 Fig in S2 Appendix).

### Fishway passage efficiency

Estimates of successful upstream passage through fishways were more variable across runs and dams than the fishway attraction and entry estimates, but were still high. Mean fishway passage efficiency was 0.978 (*SD* = 0.028, *n* = 238) across all run×year×dam combinations (S7 Fig in S2 Appendix). Run-specific means across dams were 0.989 (spring–summer Chinook Salmon), 0.955 (fall Chinook Salmon), 0.990 (Sockeye Salmon), and 0.987 (steelhead). The lowest mean was at John Day Dam for spring–summer Chinook Salmon (0.965) and steelhead (0.973), at Ice Harbor Dam for fall Chinook Salmon (0.919), and at both Bonneville and John Day (0.988) dams for Sockeye Salmon.

### Fishway passage time

This passage segment potentially included time fish spent exiting to the tailrace and re-entering fishways after their initial entry event, and longer passage times were often associated with one or more exits to the tailrace by fish. Median fishway passage times across dams ranged from 5.6 h (spring–summer Chinook Salmon and steelhead) to 8.3 h (fall Chinook Salmon) (S7 Fig in S2 Appendix). Run-specific mean times were 18.1 h (*SD* = 44.4 h, *n* = 34,020) for spring–summer Chinook Salmon, 22.4 h (59.4, 12,079) for fall Chinook Salmon, 33.8 h (24.5, 2,941) for Sockeye Salmon, and 31.2 h (196.3, 24,218) for steelhead. The highest mean and median fishway passage times were at John Day Dam for all runs except Sockeye Salmon (Bonneville Dam). About 16.9% of all fishway passage times were > 24 h and ~4.6% were > 72 h (S7 Fig in S2 Appendix).

### Dam passage efficiency

**Fig 2:**
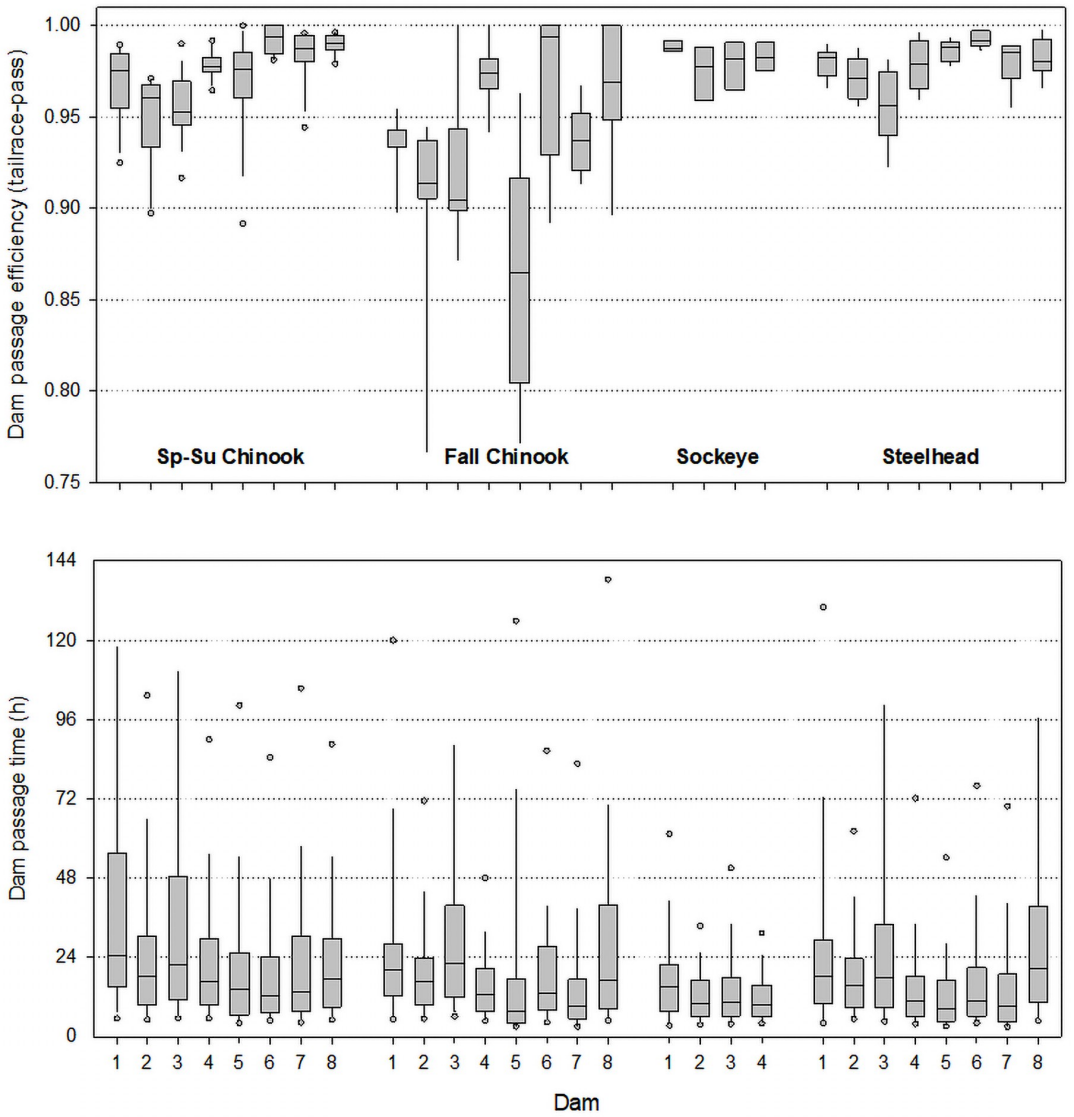
Distributions of annual dam passage efficiency estimates (top) and individual fish passage times (h) from first tailrace detection to pass a dam (bottom) for radio-tagged adult salmon and steelhead at eight study dams. Dams are ordered from downstream to upstream: Bonneville, The Dalles, John Day, McNary, Ice Harbor, Lower Monumental, Little Goose, and Lower Granite. Box plots show 5^th^, 10^th^, 25^th^, 50^th^, 75^th^, 90^th^ and 95^th^ percentiles (two 95^th^ percentile values not shown were > 144 h).

This metric captured full passage at each project, from a tailrace to a dam forebay. Mean efficiency was 0.966 (*SD* = 0.035, *n* = 245) across all run×year×dam combinations (Fig 2 and S2 Table in S1 Appendix). Run-specific means across dams were 0.975 (spring–summer Chinook Salmon), 0.933 (fall Chinook Salmon), 0.986 (Sockeye Salmon), and 0.979 (steelhead). The lowest mean passage efficiency was at The Dalles Dam for spring–summer Chinook Salmon (0.955), and Sockeye Salmon (0.983), Ice Harbor Dam for fall Chinook Salmon (0.865), John Day Dam for steelhead (0.961), and at both The Dalles and McNary dams for Sockeye Salmon (0.983).

### Dam passage time

Full-dam passage, from first fish detection in a tailrace to fish exit into a forebay, potentially included multiple movements into and out of tailraces and fishways. Median dam passage times across dams and years ranged from 15.1 h (steelhead) to 19.1 h (spring–summer Chinook Salmon) (Fig 2 and S2 Table in S1 Appendix). Run-specific mean times were 38.0 h (*SD* = 66.7 h, *n* = 37,093) for spring–summer Chinook Salmon, 34.3 h (76.7, 8,820) for fall Chinook Salmon, 16.9 h (24.2, 3,953) for Sockeye Salmon, and 50.7 h (237.3, 23,690) for steelhead. The highest mean and median times were at Bonneville Dam (spring–summer Chinook Salmon and Sockeye Salmon) and John Day Dam (fall Chinook Salmon); steelhead had their longest times at John Day Dam (mean) and Lower Granite Dam (median). About 32.9% of all full-dam passage times were > 24 h and ~9.6% were > 72 h (Fig 2).

### Passage time: Multi-dam reaches

**Fig 3:**
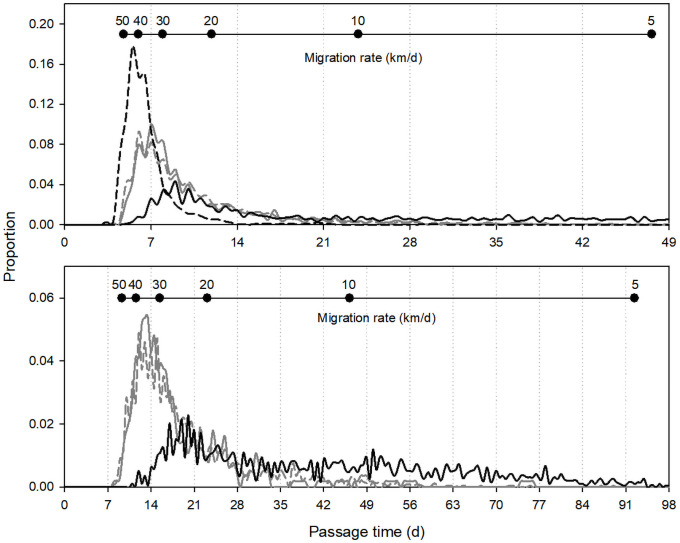
Distributions of radio-tagged salmon and steelhead passage times displayed as duration (d, x-axis) or as migration rates (inset scale, km/d) from first detection in the Bonneville Dam tailrace to last detections at top-of-ladder antennas at McNary Dam (top) and Lower Granite Dam (bottom). Spring–summer Chinook Salmon (dashed gray line), fall Chinook Salmon (solid gray line), Sockeye Salmon (dashed black line), and steelhead (solid black line). Times for steelhead temporarily entering tributaries between dams due to behavioral thermoregulation and those with overwintering behaviors are not shown, which included 23% (top) and 12% (bottom) of steelhead with calculated times.

Distributions of fish passage times from first detection in the Bonneville tailrace past McNary Dam (4 dams, three reservoirs, ~238 rkm) were right-skewed for all four runs (Fig 3). On median, Sockeye Salmon were the fastest migrants (*median* = 5.9 d, *mean* = 6.4 d, *SD* = 2.2 d, *n* = 801), followed by fall Chinook Salmon (*median* = 8.1 d, *mean* = 10.6 d, *SD* = 7.0 d, *n* = 1,437) and spring–summer Chinook Salmon (*median* = 8.3 d, *mean* = 10.3 d, *SD* = 6.4 d, *n* = 5,409). Passage times for steelhead were much longer and were far more variable (*median* = 23.9 d, *mean* = 34.6 d, *SD* = 34.6 d, *n* = 3,100) than for the other runs due to extensive behavioral thermoregulation in non-natal tributaries in summer and fall and some overwintering behavior in the Bonneville–McNary reach. On median, the time fish spent passing the four lower Columbia River dams accounted for ~32–43% of the total migration time in this reach for spring–summer and fall Chinook Salmon and Sockeye Salmon, and ~11% of the total time for steelhead.

Passage time distributions in the longer reach from the Bonneville tailrace past Lower Granite Dam (8 dams, 7 reservoirs, ~462 rkm) were also right-skewed (Fig 3). Despite a large difference in sample sizes, passage times for fall Chinook Salmon (*median* = 15.3 d, *mean* = 19.2 d, *SD* = 10.7 d, *n* = 112) were very similar to those for spring–summer Chinook Salmon (*median* = 16.2 d, *mean* = 19.6 d, *SD* = 10.3 d, *n* = 2,497). Passage times for steelhead were highly variable due to thermoregulatory and overwintering behaviors. Summary metrics were longer for all steelhead (*median* = 43.9 d, *mean* = 57.7 d, *SD* = 48.4 d, *n* = 2,143) than for the subset that did not overwinter in the Bonneville–Lower Granite reach (*median* = 39.2 d, *mean* = 43.9 d, *SD* = 24.7 d, *n* = 1,929). On median, the time fish spent passing the eight dams accounted for 39% (spring–summer Chinook Salmon), 34% (fall Chinook Salmon), and 12% (steelhead) of the total migration time through this reach (overwintering steelhead excluded from the total passage time estimate).

### Final distribution: All fish

**Fig 4:**
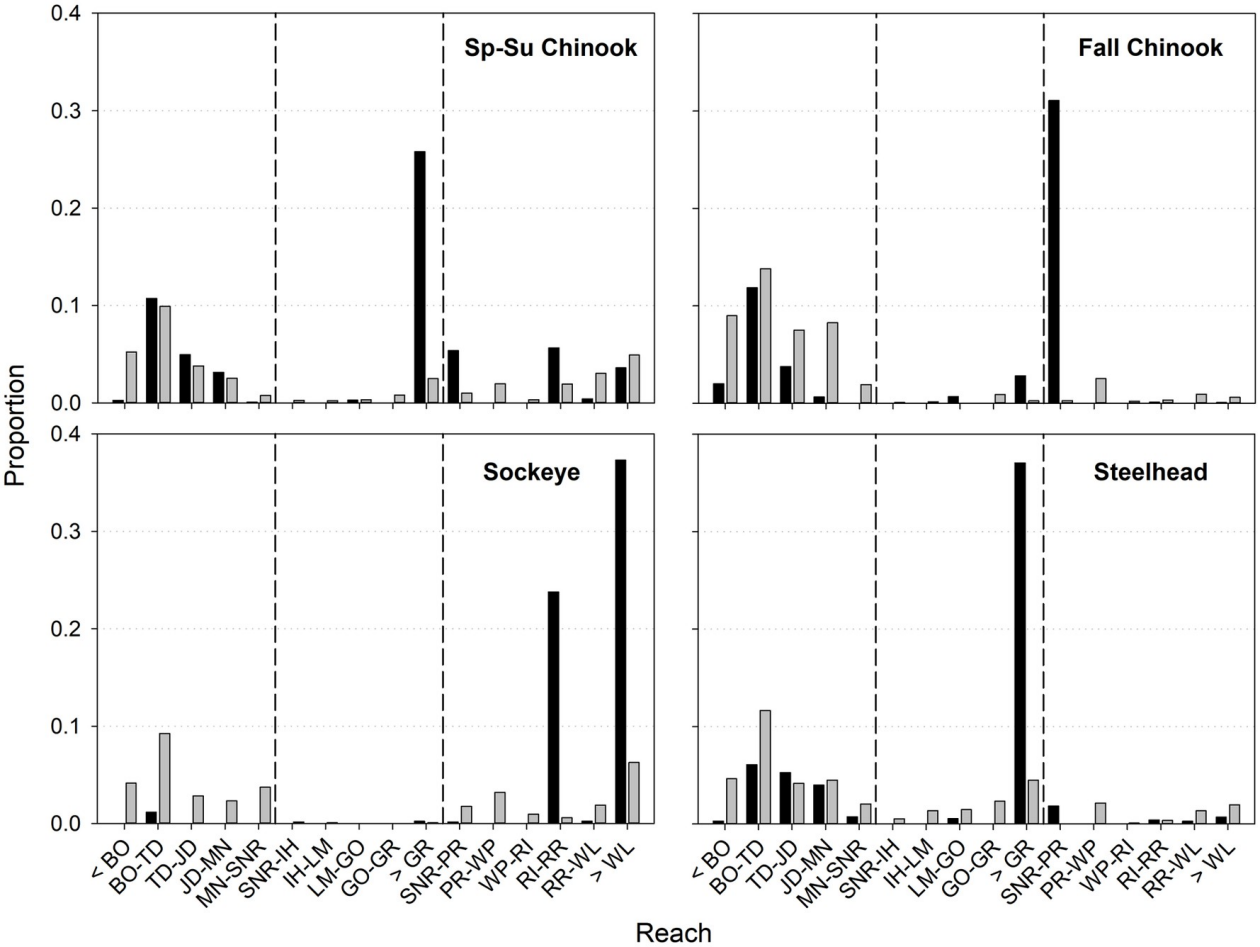
Distributions of final detection locations for radio-tagged Chinook Salmon, Sockeye Salmon, and steelhead among Columbia and Snake River reaches. Black bars represent final detections in tributaries and hatcheries and gray bars represent last detections in main stem reaches, with reported harvest and unknown fate combined. Vertical dashed lines separate the lower Columbia River (left), Snake River (SNR, middle), and mid-Columbia River (right). See Fig 1 for dam abbreviations.

Across years, the percentages of tagged fish last detected in tributaries, hatcheries, or at known main stem spawning sites were 61% (spring–summer Chinook Salmon), 53% (fall Chinook Salmon), 64% (Sockeye Salmon), and 57% (steelhead; Fig 4 and S1 Table in S1 Appendix). The remainders (~36–47%) of each run were harvested or had unknown fate in main stem reaches. Fish were distributed among almost all reaches, with notable concentrations in Snake River tributaries above Lower Granite Dam (spring–summer Chinook Salmon and steelhead), in the unimpounded Hanford Reach of the Columbia River downstream from Priest Rapids Dam (fall Chinook Salmon), and in the Wenatchee and Okanogan Rivers in the upper Columbia River basin (Sockeye Salmon). When we censored fish that were reported harvested in dam tailraces and reservoirs, the percentages of ‘successful’ migrants (i.e., presuming that harvested fish would have passed dams) were 67% (spring–summer Chinook Salmon), 67% (fall Chinook Salmon), 69% (Sockeye Salmon), and 67% (steelhead).

### Final distribution: Fish that did not pass dams

**Fig 5:**
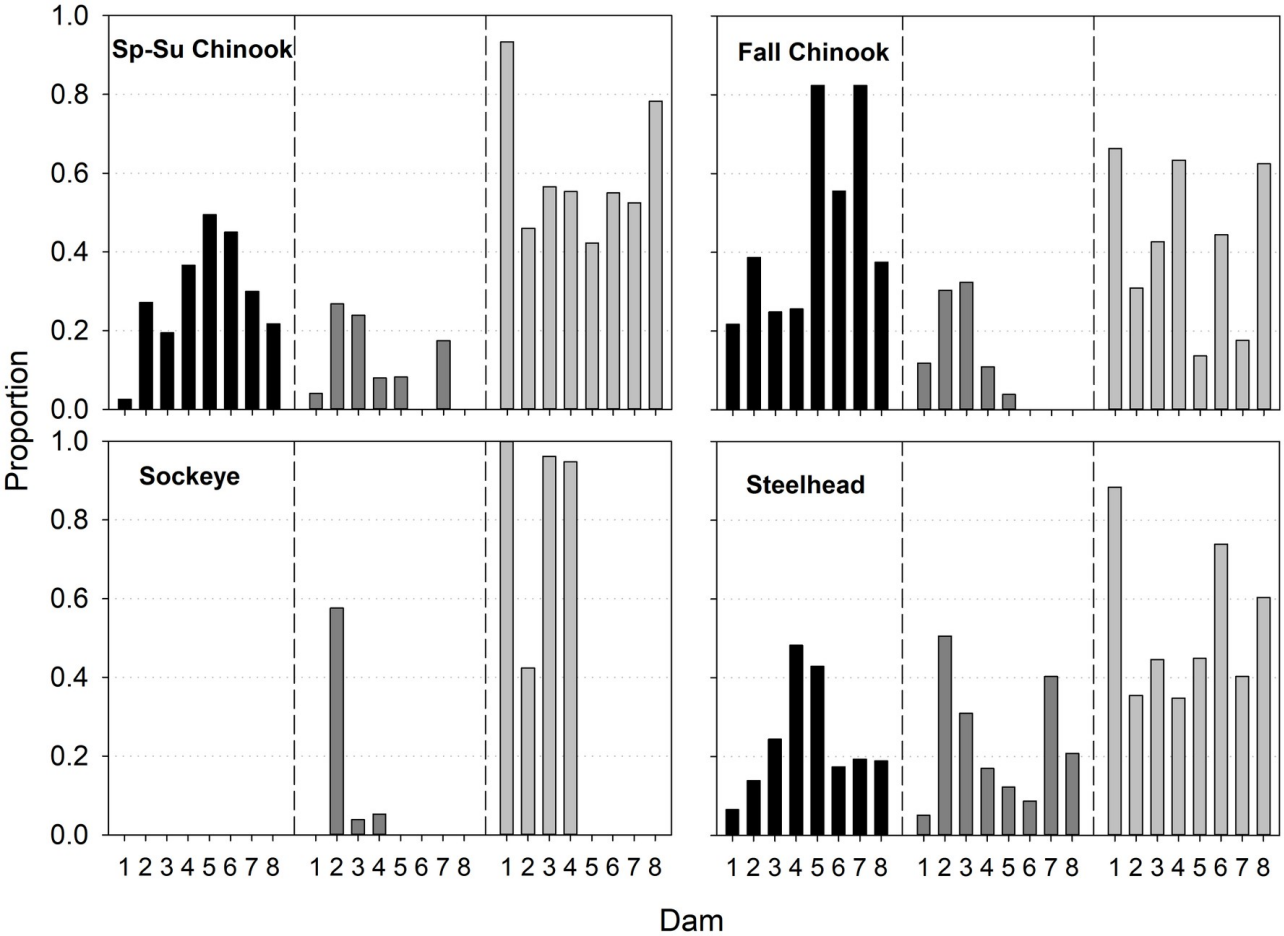
Last detection locations and estimated fate of radio-tagged salmon and steelhead that were detected at dams, but did not pass, shown as the proportion of those that did not pass at each dam. Black bars are the proportion last detected at downstream tributaries, hatcheries, or main stem spawning areas. Dark gray bars are the proportion reported harvested in downstream main stem fisheries. Gray bars are the proportion with unknown downstream fate in the main stem Columbia or Snake rivers. Dams are ordered 1–8 from downstream to upstream: Bonneville, The Dalles, John Day, McNary, Ice Harbor, Lower Monumental, Little Goose, and Lower Granite.

Across dams and years, a total of 1,398 spring–summer Chinook Salmon were detected at a dam they did not pass. About 29% of these were last detected in downstream tributaries or hatcheries, 11% were reported in downstream main stem fisheries, and 60% had unknown main stem fate (Fig 5). The percent of spring–summer Chinook Salmon that entered downstream tributaries/hatcheries was highest for those that did not pass Ice Harbor (49%) and Lower Monumental (45%) dams. Downstream main stem harvest was highest for those that did not pass The Dalles (27%) and John Day (24%) dams; and unknown fate was highest for those that did not pass Bonneville (93%) and Lower Granite (78%) dams (Fig 5).

A total of 1,113 fall Chinook Salmon were detected at a dam they did not pass: 46% were last detected in downstream tributaries or hatcheries, 11% were reported in downstream fisheries, and 43% had unknown main stem fate. Last detection in downstream tributaries, hatcheries, or the Hanford Reach spawning area was highest for those that were detected at, but did not pass, Ice Harbor (82%) and Little Goose (82%) dams (Fig 5). Downstream main stem harvest was highest for those that did not pass John Day (32%) and The Dalles (30%) dams and unknown fate was highest those at Bonneville (66%) and Lower Granite (63%) dams.

Of 845 steelhead that did not pass, 24% were last detected in downstream tributaries or hatcheries, 23% were reported in downstream fisheries, and 53% had unknown main stem fate. Downstream tributary/hatchery entry was highest for the groups at McNary (48%) and Ice Harbor (43%) dams; downstream harvest was highest for non-passers at The Dalles (51%) and Little Goose (40%) dams; and unknown main stem fate was highest for the groups at Bonneville (88%) and Lower Monumental (74%) dams (Fig 5).

There were 94 Sockeye Salmon that were detected at but did not pass a Lower Columbia River dam. Downstream main stem harvest accounted for 58% of the non-passers at The Dalles Dam, and unknown main stem fate predominated for those that didn’t pass at Bonneville, John Day, and McNary dams (Fig 5). No spawning tributaries were available to Sockeye Salmon in the study reach.

## Discussion

The extensive set of quantitative fish passage metrics in this study indicate that the adult fishways at CRB dams were generally efficient and were qualitatively effective. The upstream dam passage efficiency estimates are among the highest recorded for any migratory fish population, and their documentation fills a conspicuous gap in the recent fish passage literature. Full-project passage estimates averaged 0.966 and were strikingly consistent across runs and years. Full-project fish passage times were much more variable, with median run×year×dam estimates ranging from 5–65 h. Passage time variability can be attributed to site-specific factors, but the diverse inter- and intra-annual river conditions encountered by radio-tagged fish were certainly influential, as documented in several previous studies [e.g., 43, 55, 89, 90]. The monitored spring–summer Chinook Salmon populations encountered mean Columbia River discharge that ranged from ~3,500 m^3^/s in 2001 (a drought year) to ~11,000 m^3^/s during the near-record snowmelt runoff in 1997. Temperature exposure was similarly broad, ranging from water temperatures of ~8°C for early spring migrants to >21°C for populations migrating in late summer and early fall. Adult salmon and steelhead passage over such a broad range of river conditions, and throughout their migration seasons, meets the definition of passage effectiveness proposed by Larinier et al. [91], at least at individual dams. It remains an open question whether the passage efficiency and passage time results achieve a broader goal of biological effectiveness [e.g., 20, 25], particularly for the CRB populations that must pass multiple projects. The high passage rates observed reflect the joint effects of biological attributes of the target taxon (adult anadromous salmonids) and a nearly century-long intensive adaptive management approach to fishway design, maintenance, and improvement.

### Passage through dam tailraces

Dam tailraces can be particularly challenging environments for upstream migrants to navigate [18, 33, 92]. At the CRB dams, tailraces are characterized by high average water velocity (~0.5 to > 8.0 m/s) and turbulence [62, 63], especially in spring and summer when river discharge is high and there is typically continuous spillway discharge. Regardless of season, upstream migrants must locate the relatively low-volume fishway openings amidst the much more substantial discharge emanating from turbines and spillways. The largest CRB fishway openings, for example, are located adjacent to shorelines and spillways and discharge ~50 m^3^/s [65]. This volume is just ~0.3–0.9% of mean Columbia River discharge at The Dalles Dam [e.g., 57] during much of the adult salmon and steelhead migrations.

Despite potential fishway discovery challenges, almost all radio-tagged fish that entered a tailrace were eventually detected at one or more fishway openings. The average dam-wide fishway attraction efficiency estimate (0.985) was far higher than the average attraction estimates of 0.651 and 0.550 in the multi-species reviews by Noonan et al. [1] and Bunt et al. [22], respectively. The CRB fishway attraction estimates were also higher than comparable estimates from the salmonid-specific studies (average ~0.80) in Bunt et al. [30] and in some recent salmon studies not included in the reviews (~0.80–0.90) [34, 93]. An exception was a recent radiotelemetry study of Atlantic Salmon *Salmo salar* in Sweden by Nyqvist et al. [35], where fishway attraction efficiency estimates (≥0.97) were equivalent to the CRB estimates reported here.

Importantly, we attribute the high fishway attraction efficiency at CRB dams to the availability of multiple entry points at each dam and to the appropriate siting of the entrances. At each dam, the fishway openings with the highest discharge volume were located adjacent to shorelines and at the ends of powerhouses, taking advantage of the tendency of migrating adult salmonids to orient along river banks [e.g., 94, 95]. Additional high-volume fishway openings were located closer to mid-channel adjacent to some spillways, where they were likely to be encountered by migrants attracted to spillway discharge or orienting along velocity gradients (“seams”) along the tailrace-spillway plumes. Numerous low-volume fishway openings (e.g., orifice gates and sluice gates discharging < 2 m^3^/s, [62] were distributed along powerhouse faces, providing potential entry routes for fish attracted to turbine discharge (though some of these smaller openings were operated only intermittently and also served as exit points). Regardless of where salmon or steelhead first approached a dam, individual telemetry histories showed that many fish made cross-channel movements along the powerhouses and near spillways, virtually assuring that fish would encounter one or more fishway openings. The attraction efficiency results demonstrate how providing a multi-faceted array of fishway entrance options at a barrier may substantively increase the likelihood that fish will locate potential passage routes. The results also highlight how project-wide efficiency metrics provide information that may be different than those in assessments of individual fishways in isolation.

Most fish initially passed through tailraces rapidly (i.e., in < 3 h), but passage time distributions through this segment were often multi-modal, and some fish took several days to pass. In previous analyses, we have shown that adult salmonids exhibit strongly diel movements in CRB tailraces, with peak upstream activity typically in mid-morning associated with short passage times, arrival to the fishway during afternoon associated with overnighting at the dam, and dramatically curtailed movement at night [44, 82, 96]. The tendency for a substantial portion (e.g., 20–50%, [44]) of fish to spend a night in a tailrace before approaching a fishway explains–in part–why mean tailrace passage times were considerably longer (8–10 h per run across years and dams) than median times for all four runs. A much smaller portion of the tagged fish made significant downstream movements out of tailraces and then subsequently passed, and this wandering behavior distinct from overshoot behaviors also increased mean and variance estimates. It is important to recognize that the ‘first passage’ to the fishway metric was a poor indicator of the total time that salmon and steelhead spent holding, searching for passage routes, and entering and exiting from CRB tailraces. Brown et al. [92], for example, estimated that adult Chinook Salmon spent a cumulative median of ~21 h in the Bonneville Dam tailrace versus ~5 h inside Bonneville fishways. Similarly, Crozier et al. [44] reported that the total time Snake River Chinook Salmon spent in tailraces was longer than their time inside fishways by a factor of 3–4 as they migrated past eight CRB dams. Energetic costs of time spent in both locations is high relative to time spent in reservoirs [92] and increased passage time in tailraces may also increase predation and injury risk below Bonneville Dam for populations migrating when pinnipeds are present [49, 50].

### Fishway entry

Operational criteria at the CRB fishways include hydraulic head differentials at the openings of ~0.3 m which generate fish attraction plumes with velocities targeted at adult salmonids ranging from ~1.5–3.0 m/s [e.g., 42, 64, 97]. The highest plume velocities tend to be near the tailrace surface where they are attractive to salmonids and American Shad *Alosa sapidissima*, but not necessarily for less fusiform native species like White Sturgeon *Acipenser transmontanus* and Pacific Lamprey *Entosphenus tridentatus* that also use the CRB fishways [97, 98, 100]. Nearly all radio-tagged salmon and steelhead detected outside a fishway opening eventually entered one of the fishways at each dam, suggesting that operational criteria accommodated salmonid behaviors. Dam-wide fishway entrance efficiency across all run×year×dam combinations averaged 0.990, a far higher rate than the mean of 0.396 reported by Noonan et al. [1] for 11 reviewed studies with fishway entrance efficiency estimates.

Critically, our dam-wide entrance efficiency estimates mask considerable variability in the design and performance of individual fishway openings [e.g., 64, 97, 98] and the diversity of individual fish behaviors. For example, fish attempt rates [e.g., 82, 86] and approach-to-entry times were much higher at some sites than others. Differences were associated with the opening size, hydraulic context within the tailrace, and quality of attraction cues that the openings presented, which could fluctuate within and among years as a function of river environment and dam operations, particularly spill volume and tailrace elevation. On median, radio-tagged salmon and steelhead made 3–8 fishway approaches and 1–3 fishway entries per fish per dam, but some fish had dozens of approach and entry events at individual dams and fishways (*unpublished data*). Similarly, only minutes elapsed between fishway approach and entry events for many fish, while others spent hours to days approaching multiple fishway openings before entering. Thus, the dam-wide entrance efficiency and entry time estimates capture the collective performance of all fishway openings, but they should not be interpreted as indicating that all fishway openings were equally efficient or effective. Researchers and managers should use caution when comparing these types of dam-wide passage metrics to site-specific metrics at the CRB dams or elsewhere. Notably, recent modifications to fishway entrances at some CRB dams, such as the Bonneville Dam Cascades Island fishway entrance, have included installation of ‘keyhole’ weirs with narrow profiles near the surface to increase attraction velocity, and, in conjunction with flow-disruption devices inside the fishway, wide profiles near the bottom of the weir to reduce velocity near the fishway floor for co-migrating non-salmonid species such as Pacific Lamprey [42]. The modifications reduced maintenance compared to telescoping weirs, provided an increased range of passage conditions for fish, and were demonstrated to maintain or increase passage rates for adult salmonids [42].

### Fishway passage

The fishway passage estimates were also dam-wide, meaning fish could enter at one site, exit back to the tailrace, and then re-enter the same or a different fishway opening at each dam before passing. From ~30% to >90% of tagged fish in the 245 run×year×dam combinations exited a fishway into a tailrace one or more times (*unpublished data)*. The high average fishway passage efficiency estimate (0.978) therefore captured the collective performance of all fishways at each dam. As described above for the fishway approach and entrance metrics, dam-wide fishway passage efficiency should not be directly compared to efficiency estimates for single passage routes. Some individual fishways passed far more fish than others due to inherent differences in total fishway attraction volume, the numbers and locations of fishway openings, the bathymetry downstream from each dam, and tailrace hydraulics that affected fish approach routes. The individual telemetry histories indicated that smaller-bodied adults were periodically deterred from using some fishways (e.g., Sockeye Salmon at the north fishway at The Dalles Dam; spring–summer Chinook Salmon at Little Goose Dam) during periods of high spillway discharge and concomitant tailrace turbulence. There was also compelling evidence for population-specific attraction to individual fishways that was likely related to the cross-channel distribution of olfactory cues from natal tributaries [99].

The fishway passage metrics encapsulate fish behaviors in response to a variety of structural features and hydraulic environments inside the fishways. The initial fishway segments included the collection channels, where flow was generally deep and relatively laminar (~0.5–1.2 m/s), followed by junction pools and transition areas where one or more collection channels reached the base of the pool-and-weir fish ladders. In the pre-ladder segments, a substantial volume of water was pumped through floor and wall diffusers to maintain hydraulic head at the fishway openings. Consequently, attraction cues could be confusing, particularly in the transition areas, and several studies have described how upstream migrants from multiple species often turn around near the base of CRB fish ladders and move back downstream to the tailrace [e.g., 42, 82, 100]. In an unpublished analysis using data from several study years, we estimated that ~30–62% of adult salmonids exited to the tailraces at least once at Bonneville and The Dalles dams and ~56–85% exited at least once from the John Day Dam fishways. Large majorities of these fish subsequently re-entered fishways and passed the dams, as shown by the high fishway passage estimates. Turn-around behavior was generally much less frequent after fish entered the overflow-weir sections of fish ladders. Turbulence was high in ladders, but attraction to the submerged orifices (velocity ~2.4 m/s) and weir overflow was sufficient to attain very high weir-to-weir passage. Most radio-tagged fish also had little apparent difficulty passing fishway constrictions at counting stations or through sections of vertical-slot weirs near some ladder exits that are passage constrictions for Pacific Lamprey [e.g., 82].

Full-fishway passage times, like the fishway approach-to-entry times, incorporated a diverse set of fish behaviors. Fish that moved straight through a fishway without exiting to a tailrace passed the fishways very quickly (mostly < 4 h, *unpublished data*), whereas those that exited to a tailrace typically took several times longer to pass. Because high percentages of the tagged fish exited to tailraces, the run-specific fishway mean passage times ranged from 8–34 h across dams, and much of the time actually accrued while fish were in tailraces [e.g., 44, 92]. When compounded over multiple fishway and dams, exits to the tailrace have the potential to impose biologically meaningful migration delays. For example, a Snake River spring–summer Chinook Salmon that exited a fishway to the tailrace at all eight dams would conservatively spend ~136 h (5.7 d) from first fishway entry events to pass the dams (the sum of mean passage times), with much of that time spent in energetically demanding tailraces. In contrast, the same salmon would likely spend < 32 h (1.3 d) passing fishways if it did not exit to a tailrace at any dam. Notably, while migration rates and energetic costs of migration in the pre-dam unimpounded system are unknown, rapid movement through low-velocity reservoirs may partially compensate for slower movement in tailraces and fishways. For example, migration rates (km/d) by Sockeye and Chinook Salmon through short unimpounded reaches of the Columbia and Snake rivers are similar to rates in CRB tailrace+dam+reservoir reaches [101, 102].

We have previously described how slow passage at the CRB dams was associated with reduced survival to spawning areas for all four of the studied runs [55, 102]. However, it is challenging to disentangle whether slow fishway passage and fishway exit-to-tailrace behaviors are primarily a response to conditions near or inside the fishways or are a function of pre-existing fish conditions such as energetic status, pathogen burden, or other physiological impairments [e.g., 55, 96, 103, 104]. Given the prevalence of the fishway exiting behavior, we think that confusing hydraulic cues [e.g., 39], water temperature gradients [e.g., 41], time of day [82], olfactory cues [99], and other stimuli inside the fishways prompt at least some of the exiting behavior, though a combination of extrinsic and intrinsic mechanisms is virtually certain. Regardless, modifications that reduce fish exits from fishways into tailraces should be considered by CRB dam managers given the potential for migration delays to increase energetic demands and reduce migration success [e.g., 44, 105–107]. In our opinion, reducing fishway fallout and increasing dam passage speeds are among the few potential avenues for increasing the biological effectiveness of the CRB fishways for adult salmonids.

### Full-project passage and cumulative effects of encountering multiple dams

Full-dam passage efficiency and passage times are essentially a combination of the fishway attraction, fishway entrance, and fishway passage metrics, and all of the commentary above can be used to interpret the full-dam values. Across 245 run×year×dam combinations, the dam passage efficiency estimates averaged 0.966. Even the lowest annual point estimate (0.767 for 30 fall Chinook Salmon at The Dalles Dam in 1997) was considerably higher than the averages reported for salmonids (0.617, *n* = 31 estimates) and non-salmonids (0.211, *n* = 30) in the Noonan et al. [1] review. The latter authors’ ‘passage efficiency’ metric, defined as the proportion of all fish present at a site that entered and successfully passed a fishway, was functionally equivalent to our full-dam passage efficiency metric.

While the CRB full-project estimates are atypical when compared to those for many other species and locations, it is critical to recognize the cumulative impacts for populations that must pass a series of dams. For example, compounding the average CRB dam passage efficiency estimate (0.966) over four lower Columbia dams or eight dams (lower Columbia plus lower Snake River dams) yields multi-dam values of 0.871 and 0.758, respectively. Similarly, the product of the eight full-dam passage efficiency estimates (mean values) was 0.821 for spring–summer Chinook Salmon and 0.846 for steelhead, suggesting impacts of ~15–18% for Snake River populations with natal sites upstream from Lower Granite Dam. Such compounding is clearly a simplification given the complicating effects of fish origin uncertainty and potentially indirect effects like dam-related harvest risk or fallback mortality risk (see below). However, an accumulation of effects is inevitable for upriver populations, and the dam-specific passage efficiency estimates should be evaluated in this broader migration context.

The compounding effects of multiple passage barriers may partially explain why CRB populations with the longest freshwater migration distances and highest numbers of dams to pass have experienced precipitous population declines and were among the first to be listed as threatened or endangered in the CRB (i.e., Snake River sockeye salmon, Chinook salmon, and steelhead followed by upper Columbia River spring Chinook salmon and steelhead [36, 37, 71–75]). More generally, the effects of cumulative impacts on passage effectiveness for individuals and populations are understudied in fish movement and migration research; addressing such gaps should be a research priority given the vast numbers of barriers in river networks around the world [8–10, 23–25].

### Interpreting passage ‘failure’

Defining what constitutes dam passage ‘failure’ can be vexing, particularly when species make a mix of breeding and non-breeding movements [e.g., 108], and when species do not home to natal sites during reproductive migrations [e.g., 109, 110]. In our study, many fish entered downstream tributaries or spawning areas after detection at a dam, but uncertainty about natal origin muddles interpretation of this behavior in many cases. About a quarter of spring–summer Chinook Salmon and steelhead, and almost half of fall Chinook Salmon that did not pass a dam they visited fell into this category. If natal sites were indeed downstream for these fish, then failure to pass could be attributed to navigation or homing errors. In fact, bypassing natal tributary confluences is common for some populations as they migrate through the large CRB reservoirs, and this behavior is referred to as ‘tributary overshoot’ [87, 88, 111]. Apparent overshoot behavior by the radio-tagged fish was common at Ice Harbor Dam, where many spring–summer and fall Chinook Salmon were detected before moving downstream and re-entering the Columbia River; at Little Goose and Lower Granite dams, which were approached by many fall Chinook Salmon that eventually moved downstream to the Tucannon River or Lyons Ferry Hatchery; and at McNary Dam, where many steelhead were detected that eventually moved downstream to the John Day or Deschutes rivers. Origin was unknown for most radio-tagged fish, so it was impossible to differentiate tributary overshoot from permanent straying [e.g., 48]. An alternative explanation for overshoot behavior in adults of unknown origin is that straying could be considered a behavior of last resort for individuals seeking spawning opportunities after failing to pass a dam.

The mechanisms generating the relationship between downstream harvest and dam passage failure is somewhat more difficult to parse. Of the fish detected at a dam that they did not pass, about 11% of Chinook Salmon (both runs), 24% of steelhead, and 58% of Sockeye Salmon were reported harvested downstream. While some of the fisheries mortality may be related to overshoot fallback and increased exposure to fisheries for most populations, the fact that all Sockeye Salmon had natal origins well above the study system implies other mechanisms were at work for this species. The largest numbers of fish of all species were harvested in tribal and recreational fisheries below The Dalles and John Day dams. In the absence of fisheries, some harvested fish would almost certainly have passed the dams where they were detected, and it is therefore difficult to wholly ascribe such passage failures directly to the dams. Rather, harvest of fish after they initially entered tailraces or fishways might be considered an indirect, time-mediated effect of dams analogous to the predation mortality that occurs in dam tailraces, fishways, and forebays [e.g., 112–114]. In the CRB, such human harvest is qualitatively similar to sea lion predation on adult spring Chinook Salmon in the Bonneville Dam tailrace, where ~1–6% of the run has been consumed in recent years [50, 115]. However, much of the fisheries mortality is associated with gill net fisheries in the reservoirs of Bonneville and The Dalles dams.

Passage ‘failure’ was perhaps a slightly less ambiguous conclusion for fish that had unknown main stem fate after being detected at a dam. Across dams and runs, 40–60% of the fish that did not pass were in the unknown fate category. We (and others) have speculated that many were migration mortalities, some were harvested but not reported, and others entered spawning areas without detection [e.g., 45, 46, 102, 116]. It would be difficult, of course, to identify a causal relationship between failed dam passage attempts and any of these migration outcomes for individual salmon or steelhead.

In our most thorough statistical assessment of adult migration survival through the CRB migration corridors to date, we used time-to-event regression models with a mixture of covariates that included time-varying environmental data, individual fish traits, and fish passage times at the dams [55]. The analysis used fish radio tagged from 1996–2003 (about two-thirds of the sample included here), and concluded that fish with relatively slow passage at individual CRB dams or from Bonneville Dam past McNary Dam were less likely to be successful migrants. Several covariates affected instantaneous passage likelihood at individual dams, including: time of day (limited passage at night); water temperature (faster passage when warmer); river and spillway discharge (slower passage during higher flow); and fish body length (smaller fish tended to pass faster). These patterns provide useful information about adult salmon and steelhead behaviors at the dams and the relationship between passage rate and survival, but Caudill et al. [55] concluded that the specific mechanisms for apparent mortality at either spatial scale were unresolved.

Research progress on the passage failure question will likely require several strategies. For example, genetic stock identification and identification via parentage-based tagging [e.g., 46, 68] of future telemetered fish would dramatically reduce the uncertainties associated with fish origin. Collection of additional fish trait and risk data, including physiological or transcriptomic measures of energetic status [e.g., 117, 118], pathogen burden [e.g., 119, 120], or stress indicators [e.g., 103, 121, 122], and quantification of individual thermal experience [e.g., 41, 46, 122] or activity budgets [123], would likely yield important insights on fish behaviors at the dams and on passage failure mechanisms. Tagging and monitoring individual fish has proven effective for assessing migration survival questions over large geographic areas [e.g., 124–126], but passive monitoring methods can also provide insight on failure mechanisms. For example, video or acoustic monitoring [e.g., 100] could be used at known passage problem areas like the fishway transition areas, perhaps in conjunction with experimental modifications of fishway structures or hydraulics [e.g., 39, 95] targeting a reduction in turn-around behaviors.

### The ‘biological effectiveness’ challenge

Across runs and years, 53–64% of radio-tagged adults were last detected in dozens of tributaries, hatcheries, and potential main stem spawning sites, not accounting for fisheries take in Columbia River and Snake River reservoirs and tailraces. These estimates increased to ~67–69% when we censored fish that were reported harvested in dam tailraces and reservoirs. However, we reiterate that interpreting fisheries harvest in relation to fishway passage ‘success’ or ‘failure’ is difficult, and censored and uncensored estimates each provide some heuristic value with respect to fishway effectiveness. Spawner escapement estimates to reproductive sites in the uncensored 53–64% range may be sufficient for meeting broad fisheries management objectives, such as providing terminal-area harvest opportunities [e.g., 127] or maintaining production within metapopulations [i.e., the aggregate of individual populations, 128]. Unfortunately, the radiotelemetry dataset was not well suited for assessing survival metrics or conservation objectives for individual at-risk populations [e.g., 129, 130] because the origin of most tagged fish was unknown [but see 45, 46]. Origin uncertainty significantly complicates survival estimation when multiple geographically-distributed spawning sites are interspersed among a series of dams [e.g., 2, 116]. It was also well beyond our study scope to assess other biologically meaningful indicators of biological effectiveness. This included how reproductive success or fitness of individuals or populations was associated with prior fish behaviors at the dams and the potential for delayed effects or associations between dam passage behavior and mortality [55], including prespawn mortality in spawning areas [131, 132]. Measuring the potential delayed or cumulative effects of dam passage on fitness requires data on post-passage survival, spawning success, and survival of progeny [e.g., 93, 133]. It was not logistically possible to collect these data given the geographic scope of the study and the resources required to make such assessments, though advances in tagging technology and monitoring arrays are making such assessments increasingly feasible.

The biological effectiveness of the CRB fishways is also difficult to assess given the radical transformation of the Columbia and Snake rivers and uncertainty about realistic benchmarks. There are no historic data on adult migration survival in the undammed migration corridor, for example, though we presume that survival varied–perhaps widely–among years, seasons, river reaches, species, and populations. It is possible that the dam passage efficiency estimates and related estimates of escapement to spawning areas in this study reflect a level of adult migration survival that is comparable to, or even potentially higher than historic levels, but we think such a conclusion would be disingenuous.

Interpreting the migration rate component of biological effectiveness presents a similar set of challenges. Present day adult migrants encounter three basic habitats in the Columbia and Snake River migration corridors: the hydraulically complex and largely unnatural environments in dam fishways, deep, low-velocity reservoirs, and tailrace sections that likely resemble previously unimpounded high-gradient reaches (i.e., rapids). We have hypothesized that the relatively slow and energetically-demanding adult passage at the CRB dams may be offset, to varying degrees, by rapid and less demanding passage through reservoirs [43, 44]. This hypothesis is impossible to test directly in the absence of pre-dam migration rate and energetic cost data, though fish migration rates through the remaining unimpounded reaches in the CRB appear similar to those in tailrace+fishway+reservoir reaches [101, 102], as noted above. Future comparison of conspecific data from other regional populations in undammed large rivers [e.g., 124, 125] could be used to make some inferences. What is reasonably well understood is that adult migration rates through multi-dam CRB reaches are sensitive to river discharge and temperature [e.g., 43, 134] and that decades-long warming (and reduced discharge) in the migration corridor has led to faster migration by some populations [e.g., 89] and a mix of faster and slower migration for others [e.g., 51, 54]. Warmer conditions also raise the energetic cost of migration, and the nexus of migration speed, river temperatures, and bioenergetics has been a recent adult research focus in the CRB [e.g., 44, 106, 131, 132, 135]. Developing empirical relationships among adult passage behaviors (e.g., passage rate and efficiency) and subsequent fitness outcomes is critical to defining biologically appropriate effectiveness targets in the CRB, especially because conditions are predicted to warm in the region [e.g., 136].

### The ‘gold standard’ conundrum

The life history and physiology of Pacific salmonids provide the foundation for their generally successful upstream passage through fishways at CRB dams. Returning adults are strongly motivated by natal-site philopatry, have exceptional navigational and swimming capabilities, and benefit from the collective social cues of conspecifics during their seasonally synchronous migrations [e.g., 27, 48, 137–140]. In combination, these traits make the salmonids an ideal family to target in fishway design and engineering projects. The collaboration between biologists and engineers in the development of CRB fishways for salmonids is widely regarded as a model for fishway design, siting, and deployment. Indeed, this process should perhaps be considered a ‘gold standard’ for the development of species-specific fish passage projects, albeit a somewhat dated standard given the many technological and biological advances in fish passage science since the CRB dams were constructed. Following the advice provided in several fish passage reviews [e.g., 11, 20, 29, 133, 141, 142], we strongly recommend that planning for new fish passage facilities follow an iterative design and testing process like the CRB’s. Furthermore, fish passage considerations should be integrated at the onset of the overall project design rather than as a more expensive post-hoc reaction to fish population declines (e.g., construction cost of modification at a single entrance is ~ 2-5M U.S.$). Moreover, instead of targeting single species or families, rigorous pre-construction experiments should include as many native species, life history types, migration systems, and variation in body size and morphology as possible. The potential role of philopatry should be carefully considered. Such tests should also be conducted over a range of expected environmental conditions to increase the likelihood that new installations will be biologically effective.

Consequently, the CRB experience suggests that the pool-and-weir fishway design should not be used at new facilities without a thorough critical review. In fact, the CRB fishway design has been only modestly effective for several migratory non-salmonids within the CRB, including Pacific Lamprey [82, 97, 143], White Sturgeon [98, 144], and American Shad [e.g., 145]. Beyond the CRB, the technical pool-and-weir design (and several variants) has proven unsuitable for passing native species groups at many sites around the world [e.g., 1, 3–5, 22, 146]. Given the rush to build hydropower facilities in many developing regions [e.g., 14, 24, 147, 148], there is a temptation to deploy existing fish passage designs. However, there is a high risk that such imports will be ineffective for the diverse fish faunas in many of the targeted river systems and alternative designs [e.g., nature-like fishways; 149] or combinations of designs may be necessary to minimize impacts on the full assemblage of fish movements at a site.

In conclusion, we think that both the CRB fishway design and testing process and the existing CRB fishways are potential ‘gold standards’–at least for anadromous Pacific salmonids or other species with demonstrated high homing fidelity and swimming capacities matched to the hydraulic design. The fish passage efficiency and passage time estimates at individual dams in this study would certainly be desirable targets for migratory species at many fish passage facilities. We reiterate, however, that several conservation and management uncertainties remain for adult salmon and steelhead passage through the CRB migration corridor. These include: (1) the relationship between fish behaviors at individual dams and subsequent survival and fitness outcomes; (2) the cumulative impacts of passing multiple projects; and (3) how to best assess and improve the overall effectiveness of the fishways in the CRB hydropower system for adult migrants. Perhaps most importantly, CRB fisheries managers should strive to understand how the effectiveness of the CRB fishways varies among species and populations and whether the facilities preserve the remarkable life history diversity of Columbia and Snake River salmon and steelhead populations [e.g., 66, 150].

## Supporting information

S1 Appendix Supplementary tables: Final distributions, annual full-dam passage efficiency estimates, and annual full-dam passage times for radio-tagged salmon and steelhead. (PDF)

S2 Appendix Supplementary figures: Monitoring array detection efficiency estimates, and segment-specific fish passage efficiency estimates and fish passage times. (PDF)
