# Supplementary material for: Technical fishway passage structures provide high passage efficiency and effective passage for adult Pacific salmonids at eight large dams
Source: PLoS One. 2021 Sep 2;16(9):e0256805. doi: 10.1371/journal.pone.0256805 (PMC8412358; doi:10.1371/journal.pone.0256805)
Supplement: S1 Appendix — (PDF) [file pone.0256805.s001.pdf]

S1 Appendix: Supplementary Tables

Table S1. Final detection locations of radio-tagged salmon and steelhead, by species and run, 1996-2014. The 'Tributary / Hatchery' category is organized by river kilometer where tributaries entered the Columbia or Snake River main stem or where hatcheries were located adjacent to the main stem. The 'At a Dam' category included fish last detected at underwater radiotelemetry antennas at or inside fishways (including fishway exit sites) and those last detected at fishway PIT-tag antennas. The 'In a Tailrace' and 'In a Reservoir' categories included fish with final radiotelemetry detections and fish reported harvested at those sites. H/Hatch = hatchery; Cr = Creek

	Sp-Su Chinook		Fall Chinook		Sockeye		Steelhead	
	n	%	*N*	%	*n*	%	*n*	%
**Total release**	**12,145**	**-**	**6,134**	**-**	**1,375**	**-**	**7,832**	**-**
**Tributary / Hatchery**	**7,464**	**61.46**	**3,258**	**53.11**	**876**	**63.71**	**4,471**	**57.09**
Non-Columbia	3	0.02						
Cowlitz	2	0.02	6	0.10				
Kalama							1	0.01
Lewis	5	0.04	8	0.13			1	0.01
Willamette	5	0.04	3	0.05			5	0.06
Washougal	1	0.01	3	0.05			3	0.04
Sandy	17	0.14	8	0.13			8	0.10
Tanner Creek/BON H			94	1.53			3	0.03
Eagle Cr	4	0.03	6	0.10				
Rock Cr	10	0.08	1	0.02				
Herman Cr	2	0.02	17	0.28			17	0.22
Wind	625	5.15	27	0.44	1	0.07	54	0.69
Little White Salmon	357	2.94	184	3.00	5	0.36	122	1.56
Spring Creek Hatch			62	1.01			1	0.01
White Salmon	47	0.39	121	1.97	5	0.36	71	0.91
Hood	56	0.46	18	0.29	5	0.36	55	0.70
Klickitat	174	1.43	293	4.78			156	1.99
Fifteenmile Cr	2	0.02					1	0.01
Deschutes	586	4.83	231	3.77			412	5.26
John Day	226	1.86	5	0.08			246	3.14
Rock Cr							5	0.06
Umatilla	148	1.22	34	0.55			60	0.77
Walla Walla	9	0.07	3	0.05			57	0.73
Lyons Ferry Hatchery	4	0.03	42	0.68			34	0.43
Palouse							1	0.01
Tucannon	35	0.29					41	0.52
Clearwater	724	5.96	84	1.37			1,228	15.68
Snake above LGr	240	1.98	82	1.34	1	0.07	474	6.05
Asotin Cr							4	0.05
Grande Ronde	178	1.47	3	0.05			269	3.43
Salmon	1,826	15.03	1	0.02	2	0.15	803	10.25
Imnaha	199	1.64	1	0.02			43	0.55
Hells Canyon Dam	37	0.30	2	0.03			42	0.54
Oxbow Hatchery							4	0.05
Yakima	603	4.97	199	3.24	2	0.15	50	0.64
Hanford Reach	58	0.48	1,449	23.62	12	0.87	90	1.15

Ringold Hatchery	56	0.46	9	0.15			1	0.01
Near Ringold H	32	0.26						
Priest Rapids Hatchery	3	0.02	248	4.04			1	0.01
Wenatchee	677	5.57	7	0.11	327	23.79	32	0.41
Entiat	48	0.40			3	0.22	6	0.08
Chelan	5	0.04	2	0.03				
Wells Hatchery	102	0.84					14	0.18
Methow	158	1.30	4	0.07	4	0.29	36	0.46
Okanogan	185	1.52	1	0.02	506	36.80	18	0.23
Chief Joseph Hatchery	15	0.12			3	0.22		
Foster Cr							1	0.01
**In a Tailrace**	**897**	**7.39**	**498**	**8.12**	**100**	**7.27**	**569**	**7.27**
Bonneville	268	2.21	212	3.46	18	1.31	114	1.46
The Dalles	193	1.59	96	1.57	36	2.62	133	1.70
John Day	155	1.28	128	2.09	12	0.87	117	1.49
McNary	60	0.49	30	0.49	14	1.02	47	0.60
Ice Harbor	26	0.21	5	0.08			38	0.49
Lower Monumental	16	0.13	3	0.05			14	0.18
Little Goose	27	0.22					47	0.60
Lower Granite	20	0.16	5	0.08			24	0.31
Priest Rapids	21	0.17	13	0.21	11	0.80	1	0.01
Wanapum	1	0.01	2	0.03	1	0.07		
Rock Island	-	-			8	0.58		
Rocky Reach	2	0.02						
Wells	51	0.42	2	0.03			5	0.06
Chief Joseph	57	0.47	2	0.03			29	0.37
**At a Dam**	**2,198**	**18.10**	**627**	**10.22**	**260**	**18.91**	**1,143**	**14.59**
Bonneville	179	1.47	73	1.19	23	1.67	115	1.47
The Dalles	108	0.89	25	0.41	5	0.36	26	0.33
John Day	97	0.80	64	1.04	9	0.65	78	1.00
McNary	115	0.95	123	2.01	61	4.44	88	1.12
Ice Harbor	16	0.13			2	0.15	49	0.63
Lower Monumental	11	0.09	1	0.02	1	0.07	14	0.18
Little Goose	8	0.7	1	0.02			11	0.14
Lower Granite	404	3.33	62	1.01	1	0.07	376	4.80
Priest Rapids	227	1.87	155	2.53	43	3.13	167	2.13
Wanapum	30	0.25	11	0.18	4	0.29	4	0.05
Rock Island	184	1.52	24	0.39	9	0.65	23	0.29
Rocky Reach	257	2.12	53	0.86	13	0.95	89	1.14
Wells	562	4.63	35	0.57	89	6.47	103	1.32
**In a Reservoir**	**1,317**	**10.84**	**1,437**	**23.43**	**102**	**7.42**	**1,455**	**18.58**
Bonneville	828	6.82	688	11.22	70	5.07	702	8.96
The Dalles	223	1.84	307	5.00	17	1.24	178	2.27
John Day	130	1.07	420	6.85	4	0.29	229	2.92
McNary	13	0.11	4	0.07			90	1.15
Ice Harbor	1	0.01	6	0.10			42	0.54
Lower Monumental	2	0.02	3	0.05			53	0.68

Little Goose	7	0.06	3	0.05			33	0.42
Lower Granite	2	0.02	1	0.02			92	1.17
Priest Rapids	3	0.02	1	0.02				
Wanapum	-	-	3	0.05			1	0.01
Rock Island	12	0.10					1	0.01
Rocky Reach	51	0.42			1	0.07	13	0.16
Wells	45	0.37	1	0.02	10	0.73	21	0.27
**Release / main stem**	**269**	**2.21**	**313**	**5.10**	**37**	**2.69**	**194**	**2.48**
Release site	181	1.49	156	2.54	36	2.62	90	1.15
Below release site	88	0.72	157	2.56	1	0.07	104	1.33

Table S2. **Annual dam passage efficiency estimates** by run, year, and dam. Efficiency was calculated as the proportion of individuals detected in a dam tailrace or at any fishway antenna that eventually passed the dam. Number of fish in the denominator in parentheses. BO =Bonneville, TD = The Dalles, JD = John Day, MN = McNary, IH = Ice Harbor, LM = Lower Monumental, GO = Little Goose, and GR = Lower Granite

		Dam							
Run	Year	BO	TD	JD	MN	IH	LM	GO	GR
Sp-Su Chinook	1996	0.975 (832)	0.897 (546)	0.917 (408)	0.977 (308)	0.891 (129)	-		0.991 (106)
	1997	0.981 (968)	0.903 (790)	0.959 (656)	0.980 (598)	0.958 (331)	0.984 (316)	0.974 (308)	0.987 (297)
	1998	0.986 (942)	0.935 (814)	0.949 (668)	0.965 (593)	0.944 (267)	0.992 (247)	0.996 (243)	0.979 (240)
	2000	0.984 (966)	0.930 (903)	0.946 (717)	0.980 (636)	0.988 (248)	0.992 (244)	0.996 (238)	0.996 (235)
	2001	0.990 (865)	0.967 (1066)	0.975 (995)	0.983 (925)	0.962 (577)	0.996 (554)	0.986 (552)	0.996 (542)
	2002	0.988 (893)	0.960 (965)	0.973 (861)	0.982 (783)	0.979 (384)	1.000 (375)	0.995 (372)	0.995 (368)
	2003	0.958 (1143)	0.959 (885)	0.952 (751)	0.975 (668)	0.994 (325)	1.000 (319)	0.987 (317)	0.990 (312)
	2004	0.945 (530)	0.970 (439)	0.953 (400)	0.977 (355)	1.000 (173)	1.000 (172)	0.988 (171)	0.994 (169)
	2005	-	0.971 (139)	0.969 (131)	0.992 (122)	0.976 (42)	-	-	-
	2006	0.937 (348)	-	0.945 (218)	0.974 (196)	0.973 (111)	1.000 (108)	0.944 (108)	0.990 (102)
	2007	0.925 (293)	0.963 (409)	0.941 (372)	0.969 (324)	0.971 (170)	-	-	-
	2009	0.962 (580)	0.964 (478)	0.960 (423)	0.976 (377)	0.984 (192)	0.984 (188)	0.979 (187)	-
	2010	0.974 (580)	0.957 (462)	0.990 (409)	0.989 (371)	-	0.986 (221)	0.993 (141)	-
	2013	0.976 (589)	0.962 (524)	0.948 (465)	0.990 (413)	0.976 (166)	0.981 (160)	0.987 (156)	0.987 (154)
	2014	0.980 (588)	0.969 (408)	0.969 (446)	0.977 (399)	0.985 (196)	1.000 (192)	0.995 (188)	0.995 (185)
	**Mean**	**0.969**	**0.951**	**0.956**	**0.979**	**0.970**	**0.993**	**0.985**	**0.991**
Fall Chinook	1997	0.898 (49)	0.767 (30)	1.000 (19)	1.000 (15)	-	-	-	-
	1998	0.937 (973)	0.912 (685)	0.872 (553)	0.977 (435)	0.771 (35)	1.000 (26)	0.947 (19)	1.000 (16)
	2000	0.933 (706)	0.918 (804)	0.899 (634)	0.964 (473)	0.917 (36)	1.000 (33)	0.939 (33)	0.897 (29)
	2001	0.954 (546)	0.906 (787)	0.948 (612)	0.970 (497)	0.894 (104)	0.988 (86)	0.914 (81)	0.972 (72)
	2002	0.943 (717)	0.915 (815)	0.899 (635)	0.984 (487)	0.839 (87)	0.942 (69)	0.935 (62)	0.966 (58)
	2003	0.943 (613)	0.944 (479)	0.899 (388)	0.973 (300)	0.804 (46)	0.892 (37)	0.976 (30)	0.966 (29)
	2004	0.943 (526)	0.944 (450)	0.909 (384)	0.942 (292)	0.963 (27)	1.000 (26)	0.923 (26)	1.000 (23)
	2005	-	0.905 (464)	0.932 (367)	0.974 (272)	0.865 (37)	-	-	-
	**Mean**	**0.936**	**0.901**	**0.920**	**0.973**	**0.865**	**0.970**	**0.938**	**0.967**

Table S2 Continued.

Run	Year	BO	TD	JD	MN	IH	LM	GO	GR
Sockeye	1997	0.986 (570)	0.959 (512)	0.965 (485)	0.983 (465)	-	-	-	-
	2013	0.987 (392)	0.977 (353)	0.991 (330)	0.991 (328)	-	-	-	-
	2014	0.992 (372)	0.988 (346)	0.982 (336)	0.975 (326)	-	-	-	-
	**Mean**	**0.988**	**0.975**	**0.979**	**0.983**	-	-	-	-
Steelhead	1996	0.980 (736)	0.988 (584)	0.923 (492)	0.968 (401)	0.978 (318)	-	-	0.974 (266)
	1997	0.966 (945)	0.966 (696)	0.925 (589)	0.986 (485)	0.982 (382)	0.992 (365)	0.955 (337)	0.987 (307)
	2000	0.985 (825)	0.961 (903)	0.963 (756)	0.960 (653)	0.979 (478)	0.987 (456)	0.984 (435)	0.966 (414)
	2001	0.986 (791)	0.978 (987)	0.955 (910)	0.979 (806)	0.988 (495)	0.998 (470)	0.989 (444)	0.998 (444)
	2002	0.980 (934)	0.982 (1060)	0.954 (964)	0.963 (864)	0.992 (655)	0.992 (643)	0.981 (619)	0.977 (600)
	2003	0.990 (577)	0.971 (464)	0.957 (414)	0.974 (340)	0.989 (277)	0.996 (271)	0.989 (266)	0.981 (260)
	2004	0.983 (286)	0.956 (205)	0.972 (178)	0.994 (154)	0.990 (97)	0.989 (94)	0.989 (92)	0.978 (89)
	2013	0.966 (770)	0.982 (670)	0.982 (597)	0.996 (533)	0.985 (458)	0.998 (434)	0.969 (413)	0.990 (396)
	2014	0.984 (773)	0.971 (665)	0.978 (595)	0.991 (537)	0.994 (465)	0.989 (449)	0.986 (441)	0.995 (429)
	**Mean**	**0.980**	**0.971**	**0.956**	**0.979**	**0.986**	**0.993**	**0.980**	**0.983**

Table S3. Median full-dam passage times (h) by run, year, and dam. Number of fish in the sample in parentheses. Means-of-medians exclude annual estimates with $n < 10$ fish. BO =Bonneville, TD = The Dalles, JD = John Day, MN = McNary, IH = Ice Harbor, LM = Lower Monumental, GO = Little Goose, and GR = Lower Granite.

		Median dam passage time (h)							
Run	Year	BO	TD	JD	MN	IH	LM	GO	GR
Spring-Summer	1996	22.7 (629)	21.7 (349)	30.5 (302)	25.6 (227)	17.4 (75)	- (0)	- (0)	39.0 (64)
Chinook	1997	24.6 (905)	31.1 (556)	35.0 (531)	16.1 (327)	19.4 (276)	23.8 (289)	21.2 (264)	25.1 (281)
	1998	19.6 (846)	23.9 (530)	31.2 (505)	21.5 (358)	28.8 (235)	18.2 (194)	15.9 (161)	26.5 (163)
	2000	26.6 (895)	22.3 (555)	28.5 (492)	18.0 (465)	14.6 (241)	12.8 (216)	12.8 (208)	18.0 (179)
	2001	23.8 (703)	20.4 (832)	26.1 (692)	16.9 (599)	10.7 (485)	13.3 (354)	14.4 (406)	10.6 (413)
	2002	41.1 (749)	21.5 (704)	25.5 (711)	20.8 (612)	10.7 (369)	10.9 (372)	13.9 (307)	20.9 (303)
	2003	30.5 (954)	15.5 (614)	24.0 (640)	16.4 (449)	11.4 (215)	12.7 (297)	10.9 (220)	26.5 (218)
	2004	29.9 (438)	13.0 (342)	20.4 (331)	14.1 (251)	8.4 (166)	9.2 (170)	9.1 (132)	21.3 (163)
	2005	24.9 (78)	14.0 (115)	29.3 (117)	14.7 (56)	9.9 (6)	- (0)	- (0)	- (0)
	2006	29.1 (242)	- (0)	19.4 (179)	12.1 (74)	7.5 (4)	11.2 (96)	19.5 (94)	15.3 (95)
	2007	38.4 (223)	17.8 (224)	23.6 (304)	17.2 (142)	14.3 (72)	- (0)	- (0)	- (0)
	2009	23.0 (487)	15.4 (418)	18.2 (389)	15.8 (291)	11.5 (164)	9.5 (163)	15.9 (178)	13.8 (104)
	2010	25.5 (460)	12.1 (285)	13.9 (320)	13.3 (158)	- (0)	14.8 (195)	11.7 (80)	12.2 (72)
	2013	19.3 (456)	19.0 (352)	16.6 (397)	17.1 (223)	22.7 (67)	12.3 (143)	11.1 (149)	14.2 (146)
	2014	21.4 (451)	17.2 (392)	14.6 (382)	15.9 (286)	9.4 (128)	10.0 (191)	65.2 (19)	14.8 (178)
	**Mean**	**26.7**	**18.9**	**23.8**	**17.0**	**14.9**	**13.2**	**18.5**	**19.9**
Fall Chinook	1997	23.0 (19)	66.5 (4)	18.4 (13)	14.0 (1)	61.2 (1)	8.3 (1)	12.2 (1)	- (0)
	1998	20.6 (781)	15.7 (216)	22.8 (299)	10.1 (283)	6.7 (25)	9.8 (17)	8.4 (17)	14.8 (7)
	2000	21.8 (543)	19.2 (300)	32.2 (260)	16.5 (178)	8.2 (27)	25.0 (14)	12.4 (18)	55.3 (6)
	2001	17.1 (474)	17.6 (468)	21.4 (284)	13.9 (273)	7.5 (86)	12.4 (52)	11.8 (43)	17.6 (22)
	2002	21.0 (567)	16.7 (411)	23.4 (415)	12.8 (286)	8.6 (63)	11.1 (42)	12.4 (18)	13.1 (13)
	2003	20.3 (427)	16.1 (276)	19.1 (214)	12.0 (117)	6.0 (13)	25.3 (28)	6.4 (17)	29.3 (10)
	2004	19.8 (329)	15.4 (234)	15.8 (222)	13.0 (106)	7.3 (16)	13.0 (22)	5.0 (11)	11.6 (3)
	2005	- (0)	16.0 (51)	15.8 (144)	15.9 (23)	17.0 (9)	- (0)	- (0)	- (0)
	**Mean**	**20.5**	**16.7**	**21.1**	**13.4**	**8.8**	**16.1**	**9.4**	**20.0**

Table S3 Continued. Median dam passage times (hours) by run, year, and dam. Number of fish in the sample in parentheses. Mean values exclude annual estimates with *n* < 10 fish.

		Median dam passage time (h)							
Run	Year	BO	TD	JD	MN	IH	LM	GO	GR
Sockeye	1997	15.0 (556)	7.9 (417)	13.2 (345)	13.1 (168)	-	-	-	-
	2013	15.1 (373)	11.6 (300)	8.8 (297)	8.9 (254)	-	-	-	-
	2014	10.5 (344)	10.3 (310)	9.3 (306)	9.2 (283)	-	-	-	-
	**Mean**	**13.6**	**9.9**	**10.4**	**10.4**	**-**	**-**	**-**	**-**
Steelhead	1996	17.0 (679)	15.9 (362)	20.5 (402)	10.4 (208)	14.6 (234)	- (0)	- (0)	25.8 (158)
	1997	17.4 (794)	13.2 (300)	17.0 (479)	8.4 (305)	13.4 (351)	10.2 (269)	10.6 (196)	22.1 (211)
	2000	19.7 (748)	15.9 (656)	19.6 (516)	10.3 (375)	7.7 (418)	10.8 (315)	9.0 (272)	22.5 (236)
	2001	18.4 (737)	16.3 (819)	20.3 (509)	10.4 (424)	6.9 (406)	8.3 (293)	9.3 (239)	18.1 (208)
	2002	24.2 (829)	17.5 (759)	24.2 (685)	13.3 (523)	6.4 (558)	11.2 (541)	10.4 (357)	21.8 (212)
	2003	19.1 (484)	14.7 (362)	15.7 (333)	10.1 (123)	5.6 (117)	12.4 (242)	6.7 (123)	21.4 (137)
	2004	18.4 (246)	14.1 (153)	17.5 (147)	9.4 (89)	6.4 (72)	8.7 (86)	6.8 (60)	17.9 (63)
	2013	15.4 (399)	14.8 (404)	8.9 (381)	8.4 (103)	11.5 (77)	13.2 (302)	19.4 (1)	19.3 (237)
	2014	15.7 (523)	13.7 (378)	13.7 (329)	10.9 (166)	8.4 (82)	13.4 (316)	7.9 (318)	16.0 (253)
	**Mean**	**18.4**	**15.1**	**17.5**	**10.2**	**9.0**	**11.0**	**8.7**	**20.5**
