# Supplementary material for: Technical fishway passage structures provide high passage efficiency and effective passage for adult Pacific salmonids at eight large dams
Source: PLoS One. 2021 Sep 2;16(9):e0256805. doi: 10.1371/journal.pone.0256805 (PMC8412358; doi:10.1371/journal.pone.0256805)
Supplement: S2 Appendix — (PDF) [file pone.0256805.s002.pdf]

S2 Appendix: Supplementary Figures.

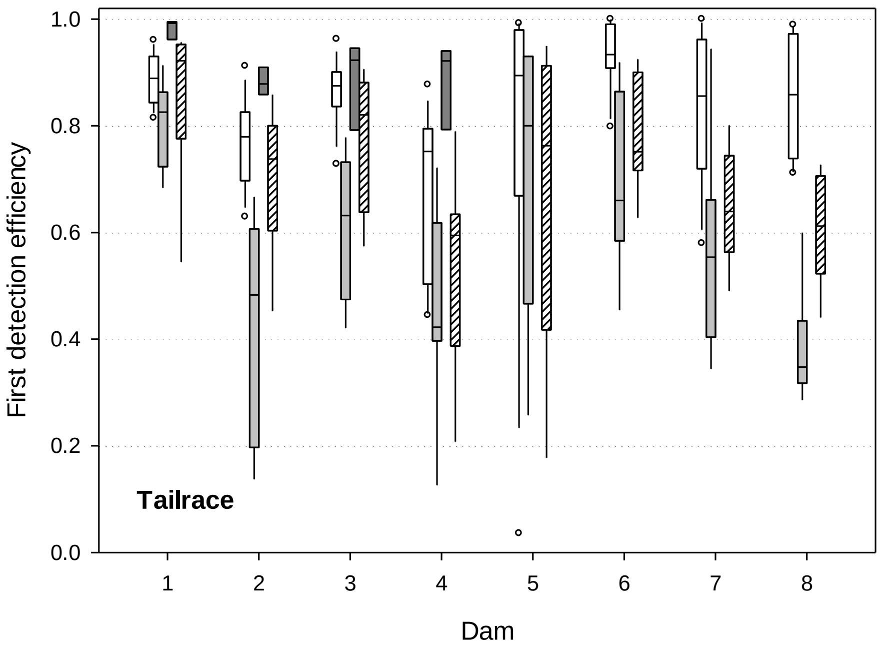

Figure S1. Box plots (5th, 10th, 25th, 50th, 75th, 90th, 95th percentiles) of the annual detection efficiency estimates for the aerial antennas used to monitor radio-tagged adult salmon and steelhead as they passed through tailraces at the study dams. Efficiency was calculated as the proportion of individuals detected at any fishway antenna that were detected at a tailrace antenna on their presumed first passage through a tailrace. Dams are ordered 1-8 from downstream to upstream: Bonneville, The Dalles, John Day, McNary, Ice Harbor, Lower Monumental, Little Goose, and Lower Granite. Open boxes = spring-summer Chinook salmon, light-gray boxes = fall Chinook salmon, dark gray boxes = sockeye salmon, hashed boxes = steelhead.

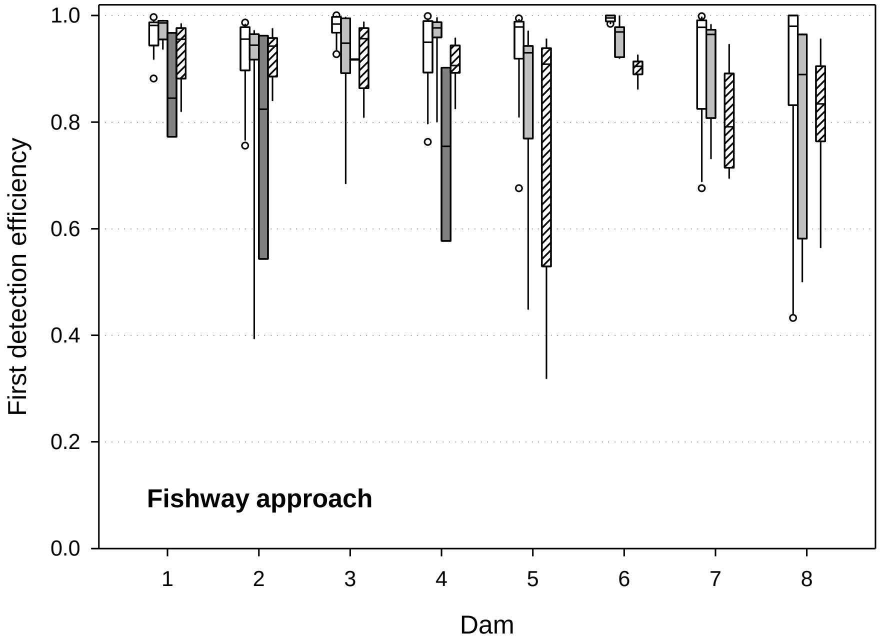

Figure S2. Box plots (5th, 10th, 25th, 50th, 75th, 90th, 95th percentiles) of the annual detection efficiency estimates for the underwater coaxial cable antennas used to monitor radio-tagged adult salmon and steelhead as they approached fishway openings at the study dams. Efficiency was calculated as the proportion of individuals detected at any fishway antenna inside a fishway that were first detected at an antenna outside a fishway opening. Dams are ordered 1-8 from downstream to upstream: Bonneville, The Dalles, John Day, McNary, Ice Harbor, Lower Monumental, Little Goose, and Lower Granite. Open boxes = spring-summer Chinook salmon, light-gray boxes = fall Chinook salmon, dark gray boxes = sockeye salmon, hashed boxes = steelhead.

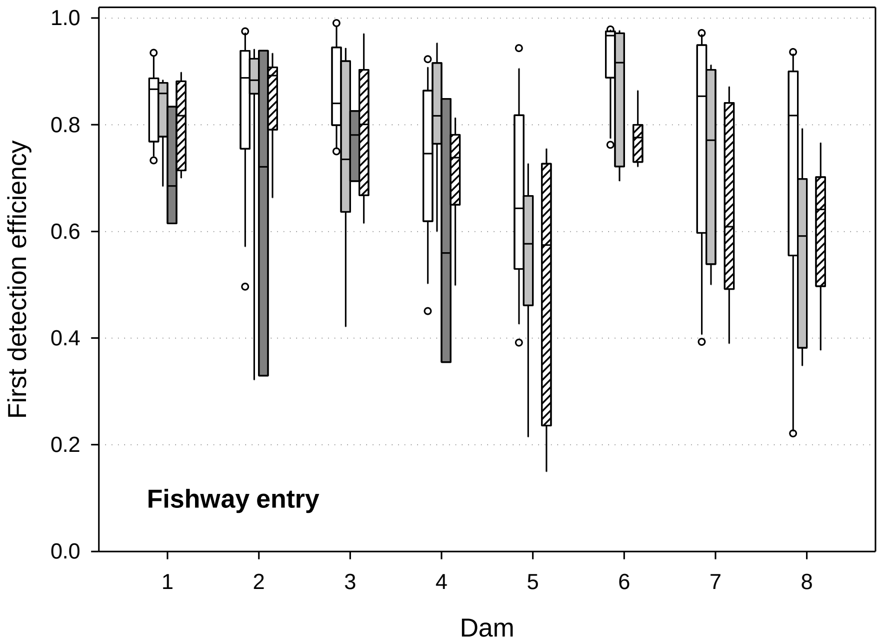

Figure S3. Box plots (5th, 10th, 25th, 50th, 75th, 90th, 95th percentiles) of the annual detection efficiency estimates for the underwater coaxial cable antennas used to monitor radio-tagged adult salmon and steelhead as they entered fishway openings at the study dams. Efficiency was calculated as the proportion of individuals detected at any fishway antenna inside a fishway upstream from a fishway opening that were first detected at an antenna just inside a fishway opening. Dams are ordered 1-8 from downstream to upstream: Bonneville, The Dalles, John Day, McNary, Ice Harbor, Lower Monumental, Little Goose, and Lower Granite. Open boxes = spring-summer Chinook salmon, light-gray boxes = fall Chinook salmon, dark gray boxes = sockeye salmon, hashed boxes = steelhead.

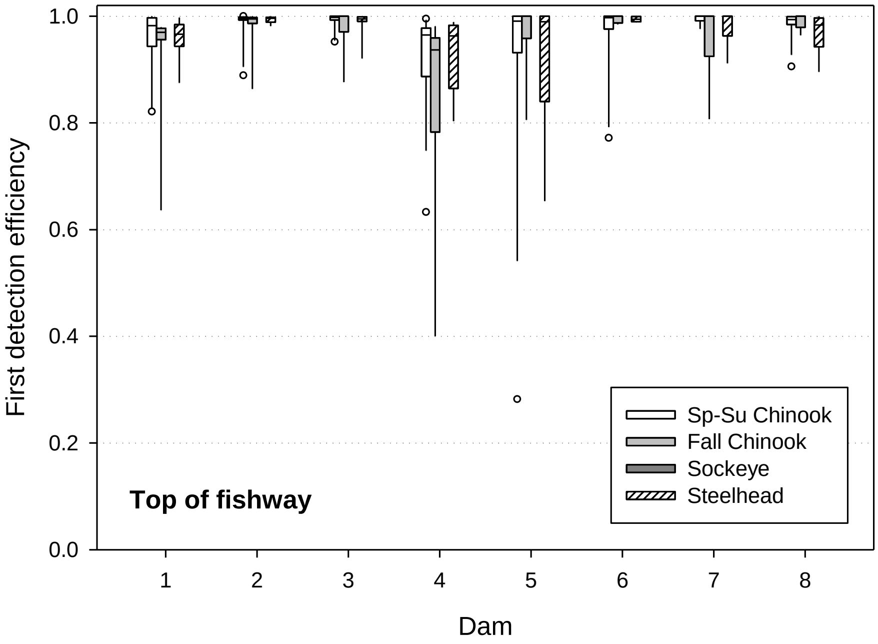

Figure S4. Box plots (5th, 10th, 25th, 50th, 75th, 90th, 95th percentiles) of the annual detection efficiency estimates for the underwater coaxial cable antennas used to monitor radio-tagged adult salmon and steelhead as they exited a fishway into a forebay at the study dams. Efficiency was calculated as the proportion of individuals detected at any monitoring site upstream from a dam that were detected at a top-of-ladder radio antenna. Dams are ordered 1-8 from downstream to upstream: Bonneville, The Dalles, John Day, McNary, Ice Harbor, Lower Monumental, Little Goose, and Lower Granite. Open boxes = spring-summer Chinook salmon, light-gray boxes = fall Chinook salmon, dark gray boxes = sockeye salmon, hashed boxes = steelhead.

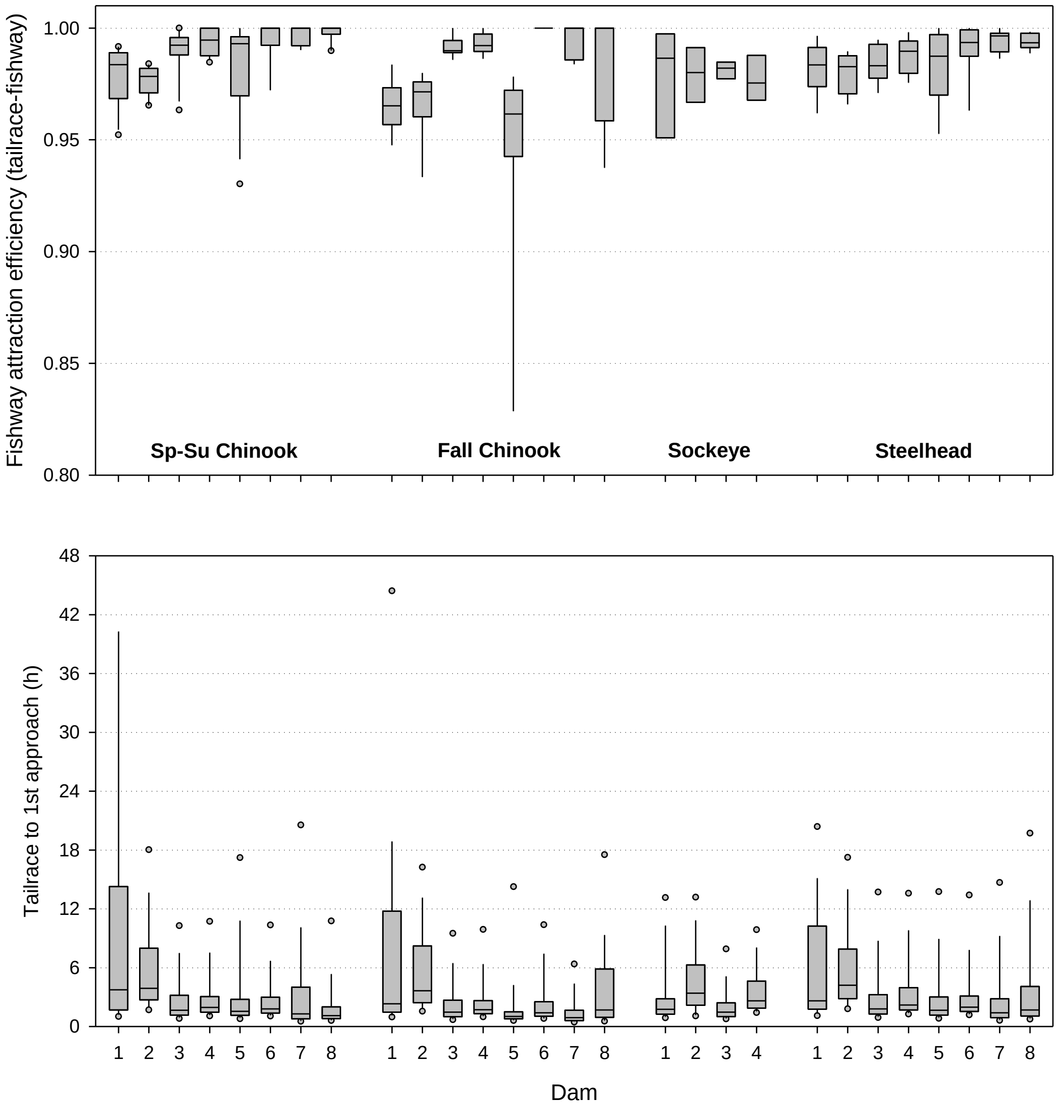

Figure S5. Distributions of annual fishway attraction efficiency estimates (top) and individual fish passage times (h) through dam tailraces to first fishway approach (bottom) for radio-tagged adult salmon and steelhead at eight study dams. Dams are ordered from downstream to upstream: Bonneville, The Dalles, John Day, McNary, Ice Harbor, Lower Monumental, Little Goose, and Lower Granite. Box plots show 5th, 10th, 25th, 50th, 75th, 90th and 95th percentiles (one 95th percentile value not shown was >72 h).

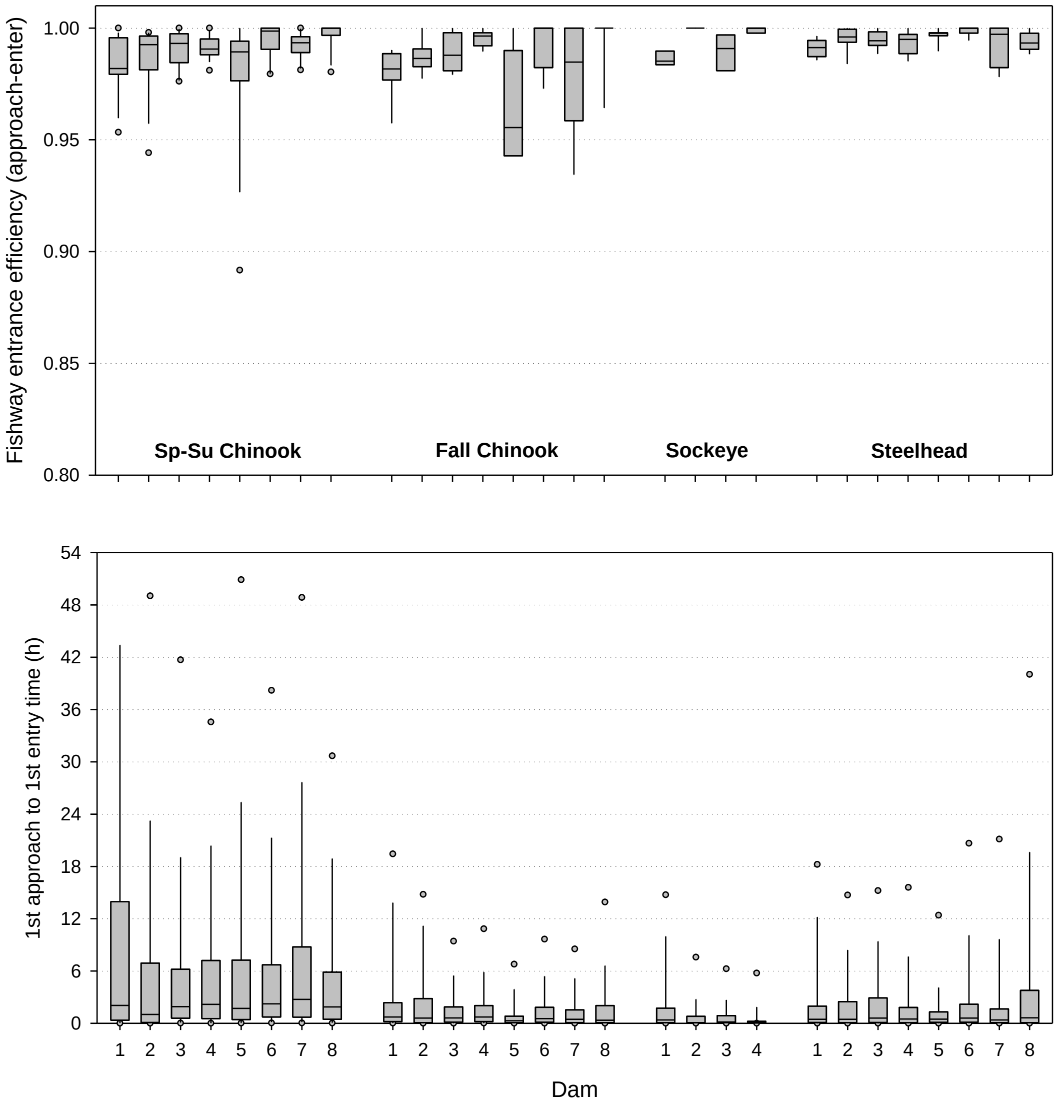

Figure S6. Distributions of annual fishway entrance efficiency estimates (top) and individual fish passage times (h) from first fishway approach to first fishway entry (bottom) for radio-tagged adult salmon and steelhead at eight study dams. Dams are ordered from downstream to upstream: Bonneville, The Dalles, John Day, McNary, Ice Harbor, Lower Monumental, Little Goose, and Lower Granite. Box plots show 5th, 10th, 25th, 50th, 75th, 90th and 95th percentiles (one 95th percentile value not shown was >72 h).

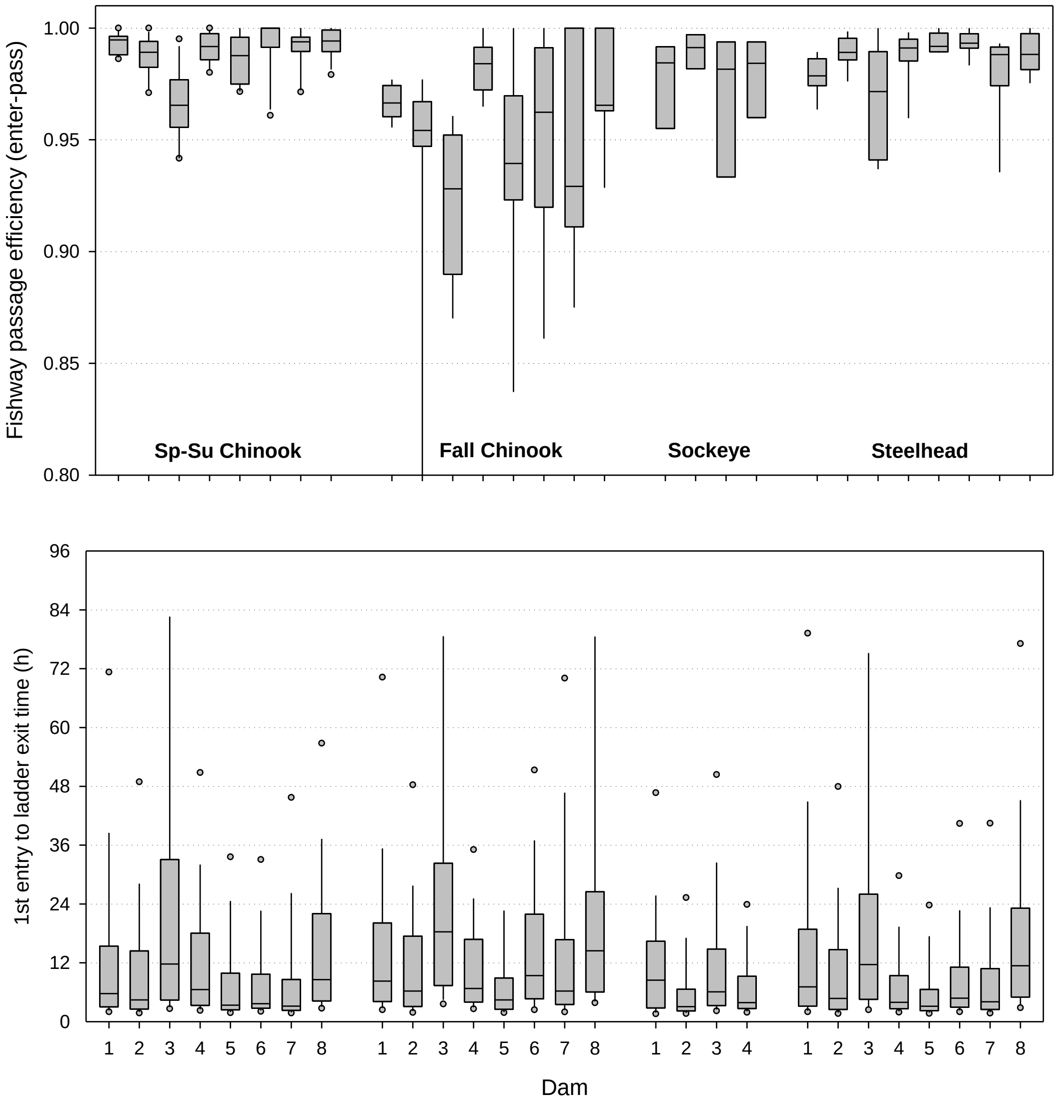

Figure S7. Distributions of annual fishway passage efficiency estimates (top) and individual fish passage times (h) from first fishway entry to pass a dam (bottom) for radio-tagged adult salmon and steelhead at eight study dams. Dams are ordered from downstream to upstream: Bonneville, The Dalles, John Day, McNary, Ice Harbor, Lower Monumental, Little Goose, and Lower Granite. Box plots show 5th, 10th, 25th, 50th, 75th, 90th and 95th percentiles (three 95th percentile values not shown were > 96 h).
